# Luteolin induces apoptosis in Philadelphia chromosome-positive acute lymphoblastic leukemia cell by regulating the PI3K/AKT signaling pathway

**DOI:** 10.3389/fphar.2025.1676034

**Published:** 2025-11-10

**Authors:** Qin Ren, Xiaobing Li, Xiangmei Ye, Leiguang Feng

**Affiliations:** Department of Laboratory Diagnostics, The First Affiliated Hospital of Harbin Medical University, Harbin, China

**Keywords:** luteolin, Philadelphia chromosome-positive acute lymphoblastic leukemia, apoptosis, network pharmacology, PI3K/AKT signaling pathway

## Abstract

**Introduction:**

Philadelphia chromosome-positive acute lymphoblastic leukemia (Ph + ALL) represents the most prevalent genetic subtype of adult acute lymphoblastic leukemia (ALL). Despite the availability of targeted therapy regimens, patients with comorbidities and older patients have poor prognoses. They are prone to relapse, necessitating the urgent identification of new safe and effective treatment options. Luteolin (LUT), a natural flavonoid compound, has demonstrated significant anticancer activity. However, its mechanism of action in the context of Ph + ALL remains poorly understood. The objective of this study was to elucidate the potential mechanisms underlying the action of luteolin in Ph + ALL.

**Methods:**

Luteolin-related targets and Ph + ALL associated targets were collected from several public databases. The intersection of these targets was then analyzed for protein-protein interactions (PPI). Additionally, we performed functional and pathway enrichment analyses employing the Gene Ontology (GO) and Kyoto Encyclopedia of Genes and Genomes (KEGG) methodologies. Core targets were selected from the PPI network, and some of these targets were further verified through cellular experiments.

**Results:**

A total of 568 luteolin targets and 1,063 Ph + ALL targets were identified, with 154 overlapping targets. The top ten targets with the highest degree values were selected as core targets, which include TP53, AKT1, ALB, TNF, JUN, IL6, EGFR, STAT3, CASP3, and BCL2. Based on GO and KEGG enrichment results, the phosphatidylinositol 3-kinase/protein kinase B (PI3K/AKT) signaling pathway was further investigated. Cell experiments demonstrated that luteolin reduced the viability of SUP-B15 cells in a time- and concentration-dependent manner. Additionally, luteolin led to an increase in reactive oxygen species (ROS) accumulation, a decrease in mitochondrial membrane potential (MMP), and a reduction in ATP content in SUP-B15 cells. At the molecular level, luteolin significantly downregulated the protein expression of p-PI3K, p-AKT, p-STAT3 and BCL-2, while upregulating the protein expression of BAX, cleaved caspase-3, and cleaved caspase-9.

**Conclusion:**

Luteolin may exert anti-Ph + ALL effects through the PI3K/AKT signaling pathway, accompanied by the regulation of other targets such as STAT3, which provides a theoretical basis for the development and screening of novel anti-Ph + ALL therapies.

## 1 Introduction

Philadelphia chromosome-positive acute lymphoblastic leukemia (Ph + ALL) is a high-risk subtype of acute lymphoblastic leukemia (ALL), with an overall incidence of 20%–25%. The incidence increases with age, accounting for over 50% of ALL cases in patients over 50 years old (Burmeister et al., 2008; Chiaretti et al., 2013). The characteristic feature of Ph + ALL is the t (9; 22) (q34; q11) translocation, which leads to the formation of the BCR-ABL fusion gene (Canichella and de Fabritiis, 2025). This gene encodes a tyrosine kinase, and these abnormally activated kinases interfere with downstream signaling pathways, resulting in enhanced cell proliferation, halted differentiation, and ultimately triggering leukemia (Kang et al., 2016; Balsat et al., 2020). Despite significant progress in the treatment of Ph + ALL in recent years, tyrosine kinase inhibitors (TKIs) targeting the BCR-ABL1 protein have become the most successful targeted therapy for Ph-positive leukemia. However, patients with infections, advanced age, and severe comorbidities have poor tolerance and are prone to relapse (Wieduwilt et al., 2021; Gaballa et al., 2022). Therefore, exploring natural-sourced compounds that possess multi-target activity and may overcome TKI resistance while exhibiting low toxic side effects has emerged as a promising new strategy in the field of Ph + ALL treatment.

**FIGURE 1:**
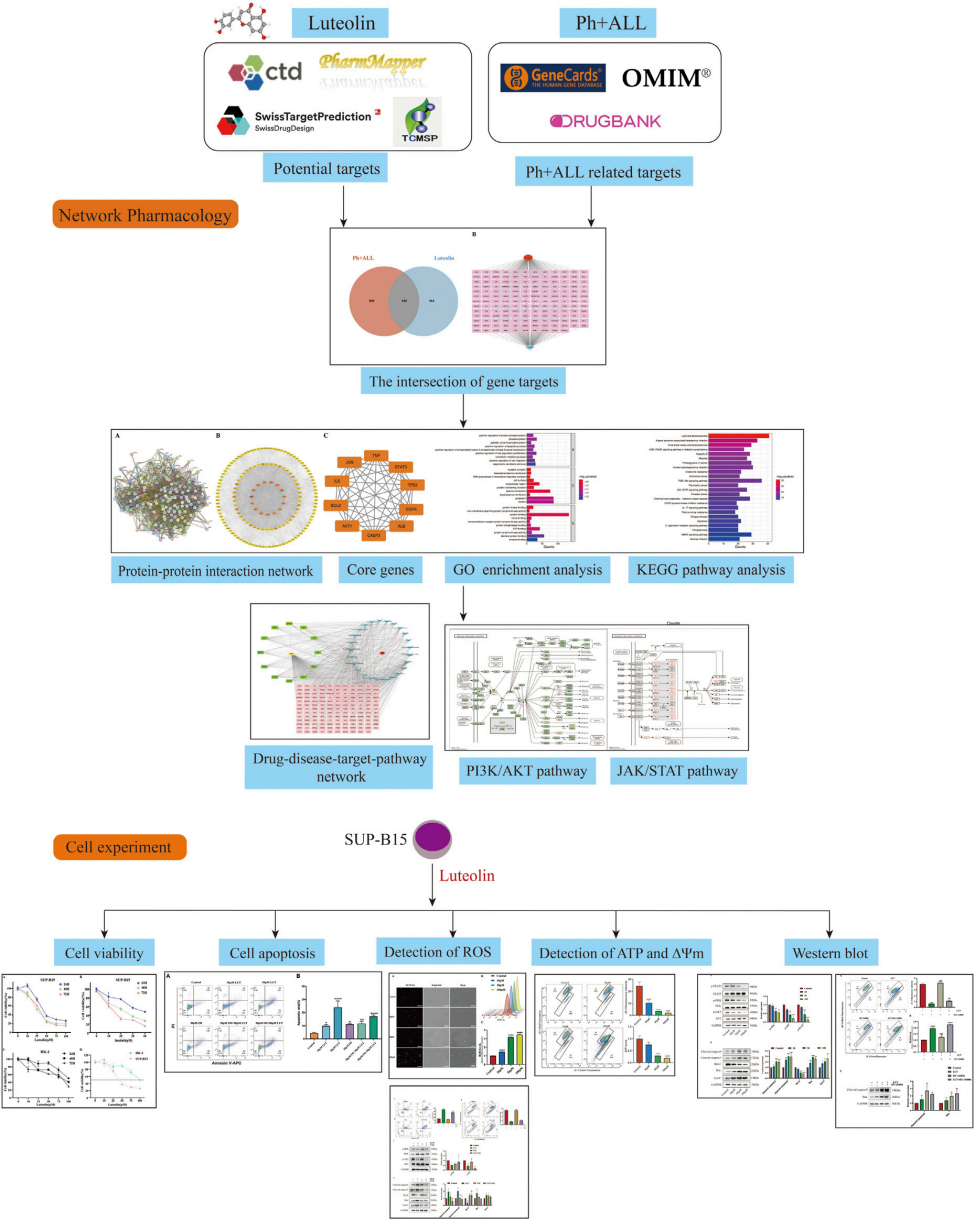
The flow chart.

Luteolin (3′,4′,5,7-tetrahydroxyflavone) is a natural compound widely distributed in plants. Due to its widespread availability, the cost of luteolin has notably decreased. Research indicates that luteolin exhibits a range of pharmacological properties, such as anti-inflammatory, antioxidant, and anticancer effects (Imran et al., 2019; Ma et al., 2023). The BCR-ABL oncogene possesses constitutive kinase activity that induces myeloid cell proliferation through various downstream signaling pathways, including the JAK/STAT pathway and the PI3K/AKT pathway (Al-Rawashde et al., 2021). Previous research has demonstrated that luteolin can inhibit cancer cell proliferation, migration, and invasion through multiple pathways, including the JAK/STAT pathway and the PI3K/AKT pathway (Singh Tuli et al., 2022). Additionally, luteolin has been found to be a promising candidate for synergistic research and may potentially reverse drug resistance in cancer cells. The combination of imatinib, a tyrosine kinase inhibitor targeting the BCR-ABL1 protein, and luteolin can act on human chronic myeloid leukemia cells K562 to reduce the dosage and toxic effects of imatinib (Danışman Kalındemirtaş et al., 2019). Thus, we hypothesize that luteolin may possess significant therapeutic potential for Philadelphia chromosome-positive acute lymphoblastic leukemia cells. This study aims to identify targets for luteolin treatment of Ph + ALL through network pharmacology screening, thereby investigating the network relationships between the drug, its targets, and related signaling pathways. Experimental validation of key targets has been conducted, providing scientific evidence for the mechanism of luteolin in treating Ph + ALL and supporting drug development. Figure 1 shows the research flow chart.

## 2 Materials and methods

### 2.1 Reagents and antibodies

Luteolin (CAS: 491–70–3, purity≥99%) and Imatinib (CAS: 152,459–95–5, purity≥99%) were acquired from MedChemExpress (Shanghai, China). The primary antibodies used include: GAPDH(P60037F), Bcl-2 (T40056F), Bax (T40051), Cytochrome C (T55734), STAT3 (T56566), p-STAT3 (T55016) were purchased from Abmart, PI3K(RC6350), p-PI3K(AP0427), AKT (A22770) were purchased from Abcolnal, p-AKT (4060T) was purchased from Cell Signaling Technology, Cleaved caspase-3(F0135), Cleaved caspase-9(F0326) were purchased from Selleck.

### 2.2 Screening of Ph + ALL and luteolin-related target genes

Luteolin-related targets were gathered from several public databases, including SwissTargetPrediction (http://www.swisstargetprediction.ch/index.php),the Traditional Chinese Medicine Systems Pharmacology (TCMSP) (https://www.tcmsp-e.com/), the Comparative Toxicogenomics Database (CTD) (https://ctdbase.org/), and PharmMapper (http://lilab-ecust.cn/pharmmapper/submitfile.html). Additionally, targets associated with Philadelphia chromosome-positive acute lymphoblastic leukemia were sourced from GeneCards (https://www.genecards.org), Online Mendelian Inheritance in Man (OMIM) (https://www.omim.org), and the Drugbank database (https://www.dru gbank.com). All target gene symbols were standardized using the Universal Protein (UniProt) database (http://www.uni-prot.org/).

### 2.3 Acquisition of drug and disease common targets

Drug and disease target data were analyzed using an online Venn diagram creation tool (http://sangerbox.com/home.html) to identify common targets for further investigation.

### 2.4 Construction of PPI networks

The common target genes of luteolin and Ph + ALL were imported into the Search Tool for the Retrieval of Interacting Genes/Proteins (STRING) online database (https://string-db.org) for PPI analysis. The species setting was configured to“*Homo sapiens*” with a confidence threshold of ≥0.40. The protein interaction network was exported and saved as a TSV file, and a visual PPI network diagram was constructed using Cytoscape 3.10.0 (Liang et al., 2022).

### 2.5 Screening of core targets

The data from the downloaded TSV file were imported into Cytoscape 3.10.0 to create a visual representation. A component-target network was then constructed, yielding a network pharmacology profile. Furthermore, topological parameters of the network were calculated using the cytoNCA plugin. Core targets were screened and identified based on the magnitude of their degree values.

### 2.6 GO and KEGG pathway enrichment analysis

Using the Database for Annotation, Visualization and Integrated Discovery (DAVID) data analysis platform (https://davidbioinformatics.nih.gov/), we performed GO enrichment and KEGG pathway enrichment analysis on the 154 overlapping targets shared between luteolin and Ph + ALL. The GO database contains biological processes (BP), cellular components (CC), and molecular functions (MF). GO and KEGG items with p-values <0.01 were selected for further research. Data visualization was performed using the online website (https://www.bioinformatics.com.cn/) (Chai et al., 2024).

### 2.7 Cell culture

The human Ph + acute lymphoblastic leukemia cell line SUP-B15 was purchased from Zhejiang Bodi Biotechnology Co., Ltd. (C5873-125, Baidi Biotech Ltd, Hangzhou, China). The cells are cultured in IMDM medium supplemented with 20% fetal bovine serum (FBS) and 1% penicillin-streptomycin, and maintained at 37 °C in a humidified incubator with 5% CO_2_ (Shi et al., 2021).

### 2.8 Cell viability assay

This study used the CCK8 assay kit (C0005, TargetMol, China) to evaluate the effect of luteolin on the viability of SUP-B15 cells. Cells in the logarithmic growth phase were selected and placed in 96-well plates at 5 × 10^4^cells/well. The experimental groups included a blank control, an untreated control group, and drug treatment groups (10, 25, 50, 75, 100 μM) with five replicates per group. Outliers (maximum and minimum values) were excluded to minimize errors. SUP-B15 cells were treated with the drug for 24, 48, and 72 h. Similarly, HK-2 cells were treated with luteolin for 24,48,72 h. Then 10 μL of CCK-8 solution was added to each well, and the cells were incubated in a culture incubator for another 2 h. The absorbance values were measured at 450 nm using a SpectraMax M5 Multi-Mode Microplate Reader (Molecular Devices, USA). The half-maximal inhibitory concentration (IC50) was calculated using nonlinear regression analysis.

### 2.9 Cell apoptosis

The cells were seeded at a density of 5 × 10^5^ cells/well into a 24-well culture plate and treated with the drug for 24 h. Apoptosis was validated using the Annexin V-APC/PI Apoptosis Detection Kit (E-CK-A217, Elabscience, China). After treatment, the cells were washed twice with pbs and resuspended in 1× Annexin V binding buffer. Subsequently, 2.5 μL of Annexin V-APC and 2.5 μL of PI staining reagents were added to the cell suspension. The mixture was incubated at room temperature in the dark for 20 min. Finally, the stained cells were analyzed using a BD FACS Lyric flow cytometer (USA). Gating Strategy: In the FSC-A vs. SSC-A scatter plot, the viable cell population was gated (Gate P1); subsequently, cell aggregates were excluded based on the FSC-H vs. FSC-A plot (Gate P2); finally, cells within Gate P2 were subjected to Annexin V-APC vs. PI analysis, and a quadrant gate was set according to the negative control and single-positive controls to distinguish between Annexin V-APC single-positive (early apoptosis), PI single-positive (necrosis), and Annexin V-APC/PI double-positive (late apoptosis/necrosis) cell populations.

### 2.10 Measurement of cellular ATP levels

Intracellular ATP levels were quantified using an ATP Assay Kit (S0026, Beyotime Biotechnology, China). Briefly, cells in the logarithmic growth phase were seeded into a 12-well culture plate at a density of 5 × 10^5^ cells per well and treated with different concentrations of luteolin (0, 30, 50, and 100 μM) for 24 h. After treatment, the cells were centrifuged, and the pellet was collected and lysed with 100 μL of ATP lysis buffer. The lysate was then centrifuged at 12,000 rpm for 5 min at 4 °C to collect the supernatant. The supernatant and the ATP standard solution were diluted with ATP dilution buffer to the desired concentrations. Subsequently, 20 μL of each diluted sample or standard was mixed with 100 μL of the reaction working solution in a black 96-well plate, with five replicates per group. Fluorescence was measured using a SpectraMax M5 Multi-Mode Microplate Reader (Molecular Devices, USA).

### 2.11 Determination of reactive oxygen species

Intracellular ROS levels were quantified using a commercial ROS assay kit (G1706, Servicebio, China). SUP-B15 cells in the logarithmic growth phase were seeded into a 12-well plate at a density of 5 × 10^5^ cells per well and treated with various concentrations of luteolin (0, 30, 50, and 100 μM) for 24 h. Following treatment, the cells were incubated with 20 μM of the fluorescent probe 2′,7′-dichlorodihydrofluorescein diacetate (DCFH-DA) at 37 °C for 30 min in the dark. The fluorescence intensity of the stained cells was then determined using a BD FACS Lyric flow cytometer (USA) and observed under a Leica DMI3000 fluorescence microscope (Germany). Data from flow cytometry and fluorescence microscopy were analyzed using FlowJo software and ImageJ software, respectively.

### 2.12 Measurement of mitochondrial membrane potential (MMP)

Changes in the MMP were detected using the Enhanced Mitochondrial Membrane Potential Assay Kit (C2003S, Beyotime Biotechnology, China). SUP-B15 cells in the logarithmic growth phase were seeded into a 12-well culture plate at a density of 5 × 10^5^ cells per well and treated with different concentrations of luteolin (0, 30, 50, and 100 μM) for 24 h. After treatment, the cells were collected and resuspended in JC-1 staining working solution, followed by incubation at 37 °C for 30 min. The cells were then washed and resuspended in JC-1 staining buffer for analysis. The fluorescence of the samples was analyzed immediately using a BD FACS Lyric flow cytometer (USA), and the resulting data were processed with FlowJo software.

### 2.13 RT-qPCR

SUP-B15 cells in the logarithmic growth phase were seeded at a density of 5 × 10^5^ cells/well into a 12-well culture plate and treated with different concentrations of Luteolin (0, 30, 50, 100 μM) for 24 h. RNA was isolated using Trizol reagent (B610409, Sangon Biotech, China) according to the manufacturer’s protocol, and the purity and concentration of RNA were detected using a UV spectrophotometer (Nanodrop 2000, USA). The quantified samples were used as templates to synthesize cDNA using a reverse transcription system (RK20429, ABclonal, China). Using cDNA as the template, amplification was performed using targeted primers (Supplementary Table S1). The PCR reaction conditions were as follows: 95 °C for 3 min, followed by 40 cycles, each cycle consisting of 95 °C for 15 s and 56 °C for 1 min. GAPDH expression was used as an internal control.

### 2.14 Western blot

SUP-B15 cells in the logarithmic growth phase were seeded at a density of 2 × 10^6^ cells/well into a 6-well culture plate and treated with different concentrations of Luteolin (0, 30, 50, 100 μM) for 24 h. Total protein was extracted from cells using RIPA lysis buffer (P0013B, Solarbio, USA) and a mixture of protease inhibitors and phosphatase inhibitors (K1007, K1015, APE × BIO, USA), and protein concentration was quantified using a BCA assay kit (ZJ102, Epizyne Biotech, China). The denatured protein samples were separated by SDS-PAGE and transferred to a PVDF membrane (IPVH00010, Millipore, Germany) and blocked with rapid blocking solution (MA0406, Meilunbio, China) for 15 min. Primary antibody incubation occurred overnight at 4 °C, followed by secondary antibody application for 1h at ambient temperature. Detection was performed using an automated chemiluminescence imaging platform (Tanon-5200, China). Gray-scale analysis was conducted using ImageJ. The experimental process was independently repeated three times.

### 2.15 Statistical analysis

Experimental data underwent statistical analysis utilizing GraphPad Prism version 9.1.0. Each experiment was performed in triplicate, with outcomes presented as mean ± standard deviation (SD). Group comparisons were made utilizing one-way ANOVA, with statistical significance established at p < 0.05 (*p < 0.05, **p < 0.01, ***p < 0.005, ****p < 0.0001; ns means no significance).

## 3 Results

### 3.1 Common targets of luteolin and Ph + ALL

**FIGURE 2:**
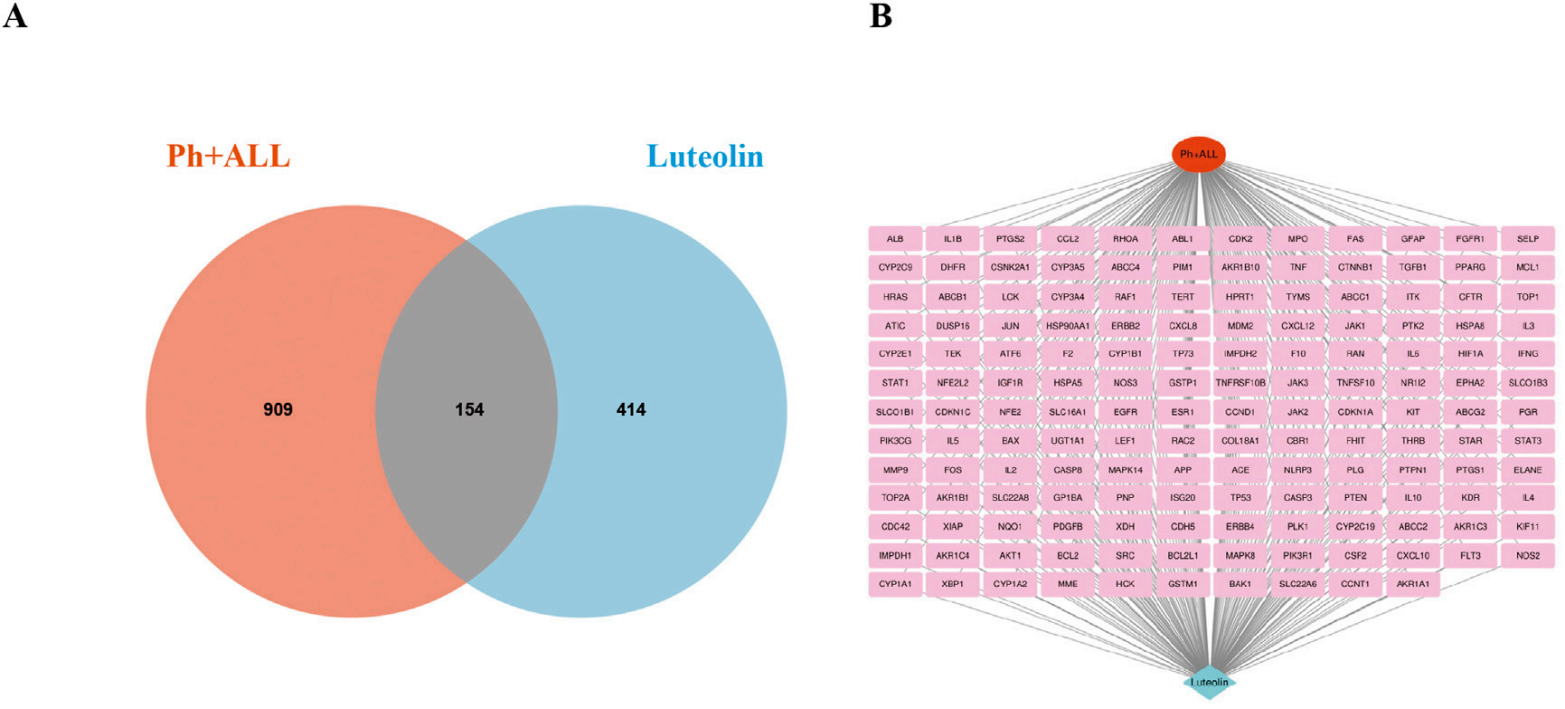
Intersection target map. **(A)** Veen diagram of target intersections of Ph + ALL and luteolin. **(B)** C-D-T network: the red module represents Ph + ALL, blue module represents Luteolin, pink modules represent common targets.

After collecting and summarizing information from multiple databases and removing redundant information, a total of 568 potential targets related to luteolin (Supplementary Table S2) and 1,063 targets corresponding to Philadelphia chromosome-positive leukemia (Supplementary Table S3) were obtained. Following a cross-analysis using a Venn diagram (Figure 2A), 154 common targets associated with both luteolin and Philadelphia chromosome-positive leukemia were identified (Figure 2B).

### 3.2 Construction of PPI network and identification of core targets

**FIGURE 3:**
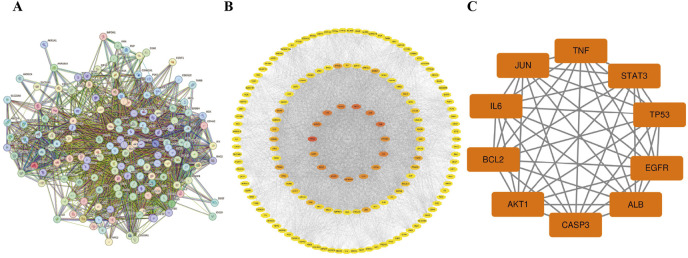
The PPI network. **(A)** The PPI network of the common targets. **(B)** PPI network of Luteolin in the treatment of Ph + ALL, the darker the color, the more critical the node is in the network. **(C)** The core genes of luteolin in the treatment of Ph + ALL.

A PPI network consisting of 154 nodes and 3,353 edges was constructed using the String database (Figure 3A). The color intensity of the nodes is proportional to their degree values. Topological analysis was performed using the CytoNCA plugin in Cytoscape 3.10.0 software (Figure 3B). The top 10 nodes with the highest degree values were selected as core targets, including TP53, AKT1, ALB, TNF, JUN, IL6, EGFR, STAT3, CASP3, and BCL2 (Table 1; Figure 3C).

**TABLE 1:** Core target information.

Gene name	Degree
TP53	238
AKT1	220
ALB	220
TNF	208
JUN	206
IL6	204
EGFR	202
STAT3	202
CASP3	198
BCL2	196

### 3.3 GO and KEGG pathway enrichment analysis

**FIGURE 4:**
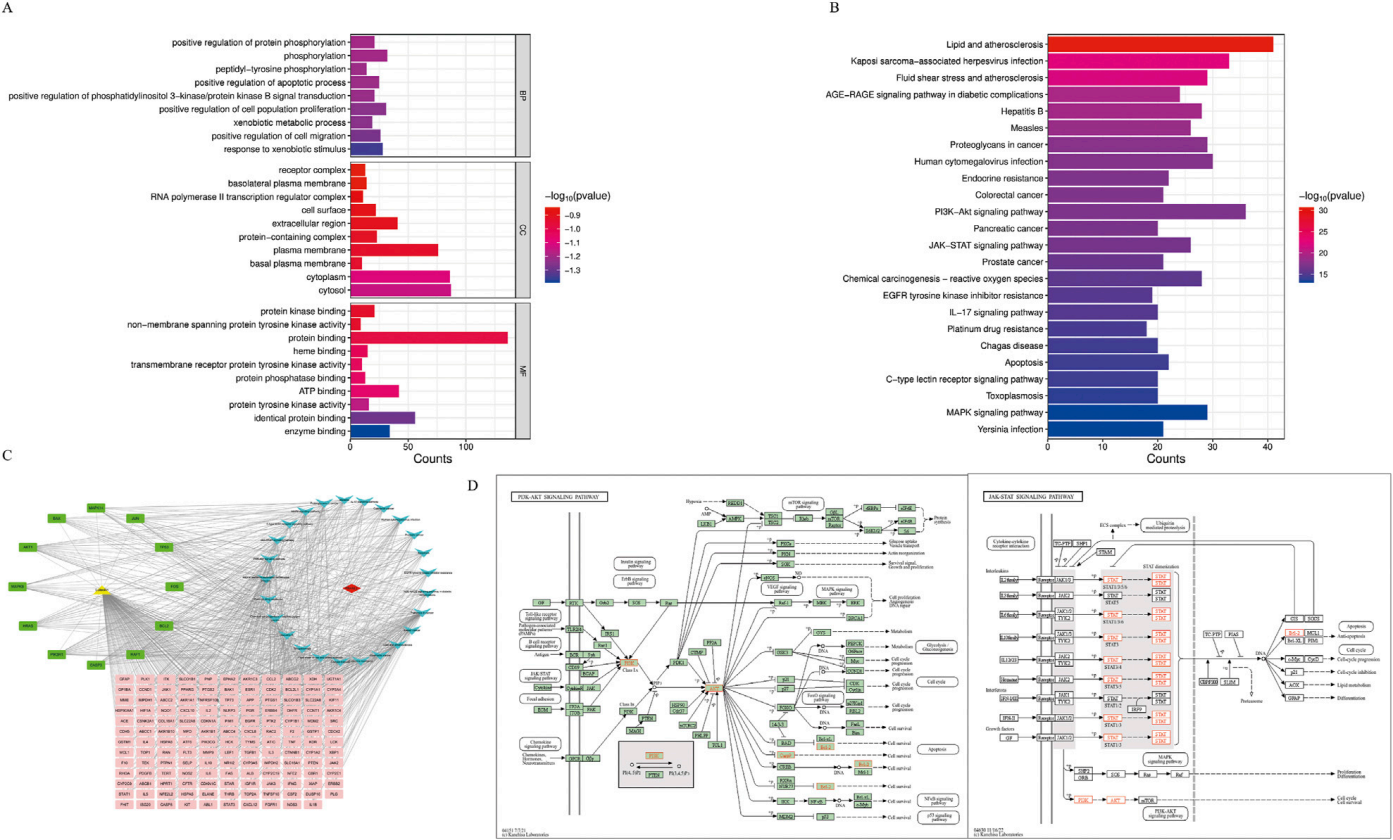
GO and KEGG pathway analysis. **(A)** The Gene Ontology Enrichment analysis: the larger the plot, the greater the number of enriched targets, the smaller the p-value, the darker the plot color. **(B)** The KEGG pathway enrichment analysis: the top 25 significantly enriched pathways. **(C)** The drug-disease-target-pathway network. The yellow module means luteolin, red module means Ph + ALL, pink and green modules mean common targets, green modules indicate greater importance in the common targets, blue modules mean signaling pathways. **(D)** The font marked in red represents the target in the PI3K/AKT signal pathway and JAK/STAT signal pathway closely related to treating Ph + ALL with luteolin.

The intersection targets were entered into the DAVID database for GO and KEGG pathway enrichment analysis. Based on p < 0.01, the top 10 GO terms were selected in BP, CC, and MF (Figure 4A), and the top 25 pathways were selected in KEGG (Figure 4B). BP terms were primarily enriched in positive regulation of protein phosphorylation, phosphorylation, and peptide tyrosine phosphorylation. CC terms were primarily enriched in the plasma membrane, extracellular region, and cell surface. MF terms were primarily enriched in protein binding, protein kinase binding, and ATPase binding. Then, the drugs, diseases, intersection targets, and 25 pathways were imported into Cytoscape 3.10.0 software to construct the drug-disease-target-pathway network (Figure 4C). The results showed that luteolin can affect multiple targets and pathways in Ph + ALL. We have focused our research on the top-ranked pathways, such as the PI3K/AKT signaling pathway and the JAK/STAT signaling pathway, and these pathways are closely related to cell proliferation and apoptosis. (Figure 4D).

### 3.4 Luteolin inhibits the proliferation of SUP-B15 cells *in vitro*

**FIGURE 5:**
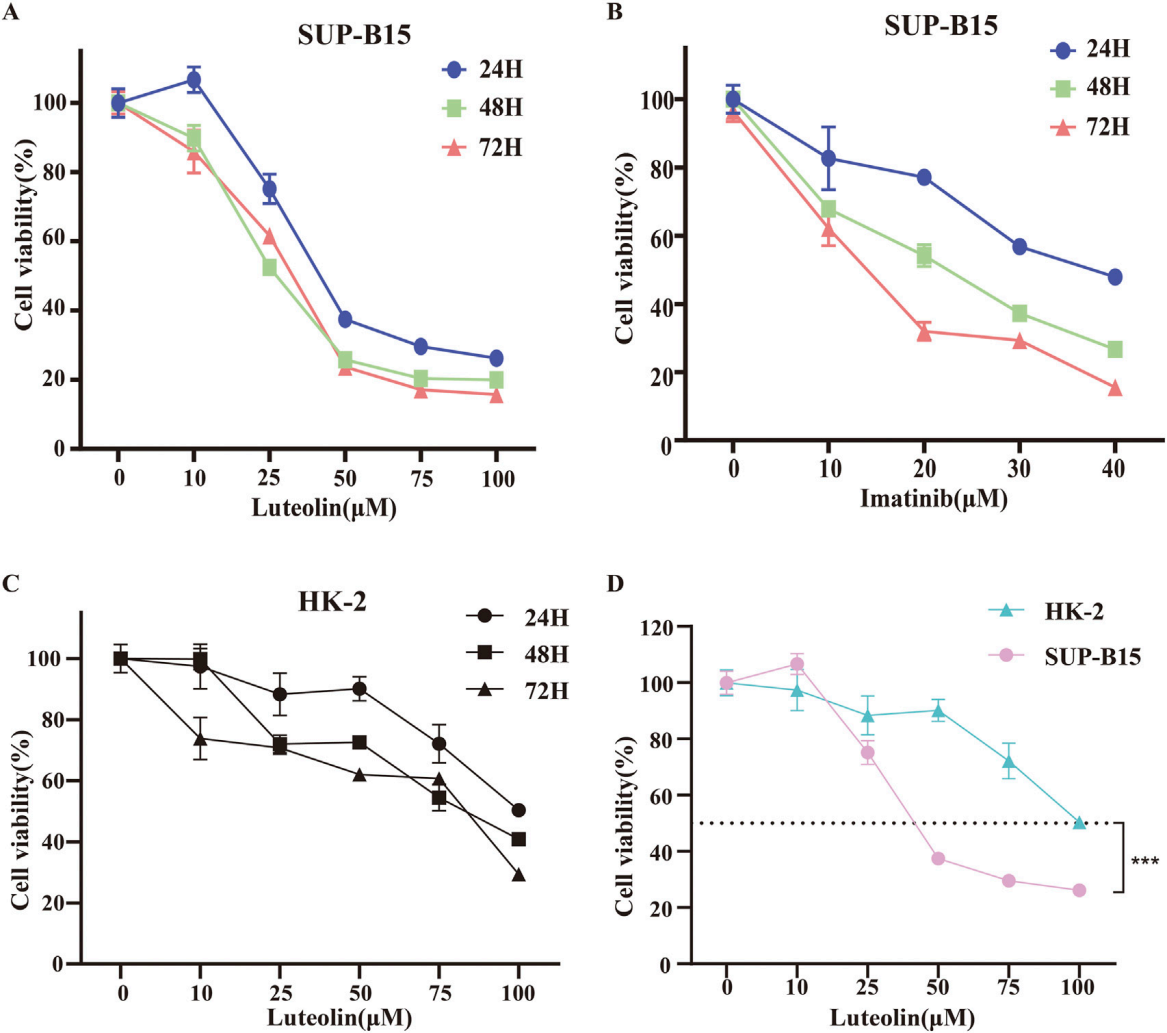
Effects of drug on cell viability. SUP-B15 cells were incubated at different concentrations of luteolin **(A)** and imatinib **(B)** for 24, 48 and 72 h, and the cell viabilities were measured via the CCK-8 assay. IC50 values were defined by the concentration that inhibited growth by 50%. **(C)** The CCK-8 assay was used to determine the viability of HK-2 cells treated with different concentrations of luteolin. **(D)** Comparison of IC50 values for SUP-B15 and HK-2 cells treated with different concentrations of luteolin for 24 h. Compared with the control group, *p < 0.05, **p < 0.01, ***p < 0.005, ****p < 0.0001.

To investigate the anticancer effects of luteolin, SUP-B15 cells were treated with different concentrations of luteolin (0, 10, 25, 50, 75, 100 μM) and the Philadelphia chromosome-positive leukemia targeted drug imatinib (0, 10, 20, 30, 40 μM) for 24, 48, and 72 h. The CCK-8 assay results showed that the proliferation of SUP-B15 cells was significantly inhibited in a time- and dose-dependent manner (Figure 5A,B). The IC_50_ values of luteolin at 24, 48, and 72 h were 27.6 ± 2.01μM, 19.21 ± 6.04μM, and 24.37 ± 4.67μM, respectively., and the IC50 values of imatinib at 24, 48, and 72 h were 32.18 ± 5.67μM, 17.3 ± 10.86μM, and 10.33 ± 5.23μM, respectively. To test the toxicity of luteolin, human normal renal tubular epithelial cells HK-2 were treated with luteolin, and the IC50 values of luteolin at 24, 48, and 72 h were 94.01 ± 8.43 μM,57.54 ± 23.19 μM,43.63 (Figure 5C), which was higher than that of SUP-B15 cells (Figure 5D). These data indicate that luteolin can inhibit the proliferation of human Philadelphia chromosome-positive leukemia cells and has low toxicity to normal human cells.

### 3.5 Effect of luteolin on the apoptosis rate of SUP-B15 cells

**FIGURE 6:**
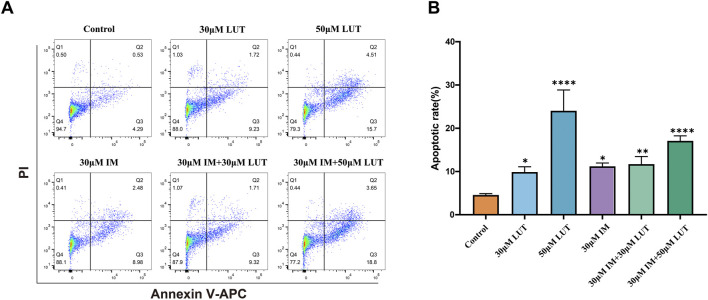
Effect of drug on apoptosis of SUP-B15 cells. **(A)** SUP-B15 cells were treated with either luteolin (30μM, 50 μM) or imatinib (30 μM) alone, or luteolin and imatinib combined for 24 H, the cell apoptosis was analyzed by flow cytometry. **(B)** Quantitative analysis of apoptotic cells. The percent-age of apoptotic cells was represented by a bar diagram. Compared with the control group, *p < 0.05, **p < 0.01, ***p < 0.005, ****p < 0.0001.

To elucidate the potential mechanisms behind the observed growth inhibition, apoptosis in SUP-B15 cells was evaluated by Annexin V-APC/PI double staining after 24 h of luteolin treatment (Figure 6A). Luteolin treatment significantly induced apoptosis in a concentration-dependent manner. Compared to the negative control (4.82%), the apoptosis rates increased to 10.95% and 20.21% at 30 and 50 μM, respectively (Figure 6B). The positive control, 30 μM imatinib, resulted in an apoptosis rate of 11.46%. Furthermore, combination treatments of imatinib with 30 or 50 μM luteolin yielded apoptosis rates of 12.1% and 22.45%, respectively. All these effects were statistically significant (P < 0.05). Notably, under the concentration combination used (30 µM luteolin +30 µM imatinib), the apoptosis rate induced by the combination therapy (12.1%) was lower than that of 50 µM luteolin monotherapy (20.21%) and slightly higher than that of 30 µM imatinib monotherapy (11.46%). This finding suggests that the interaction between the two drugs is complex and does not manifest as a simple additive effect under all conditions.

### 3.6 Luteolin treatment reduces ROS accumulation in SUP-B15 cells

**FIGURE 7:**
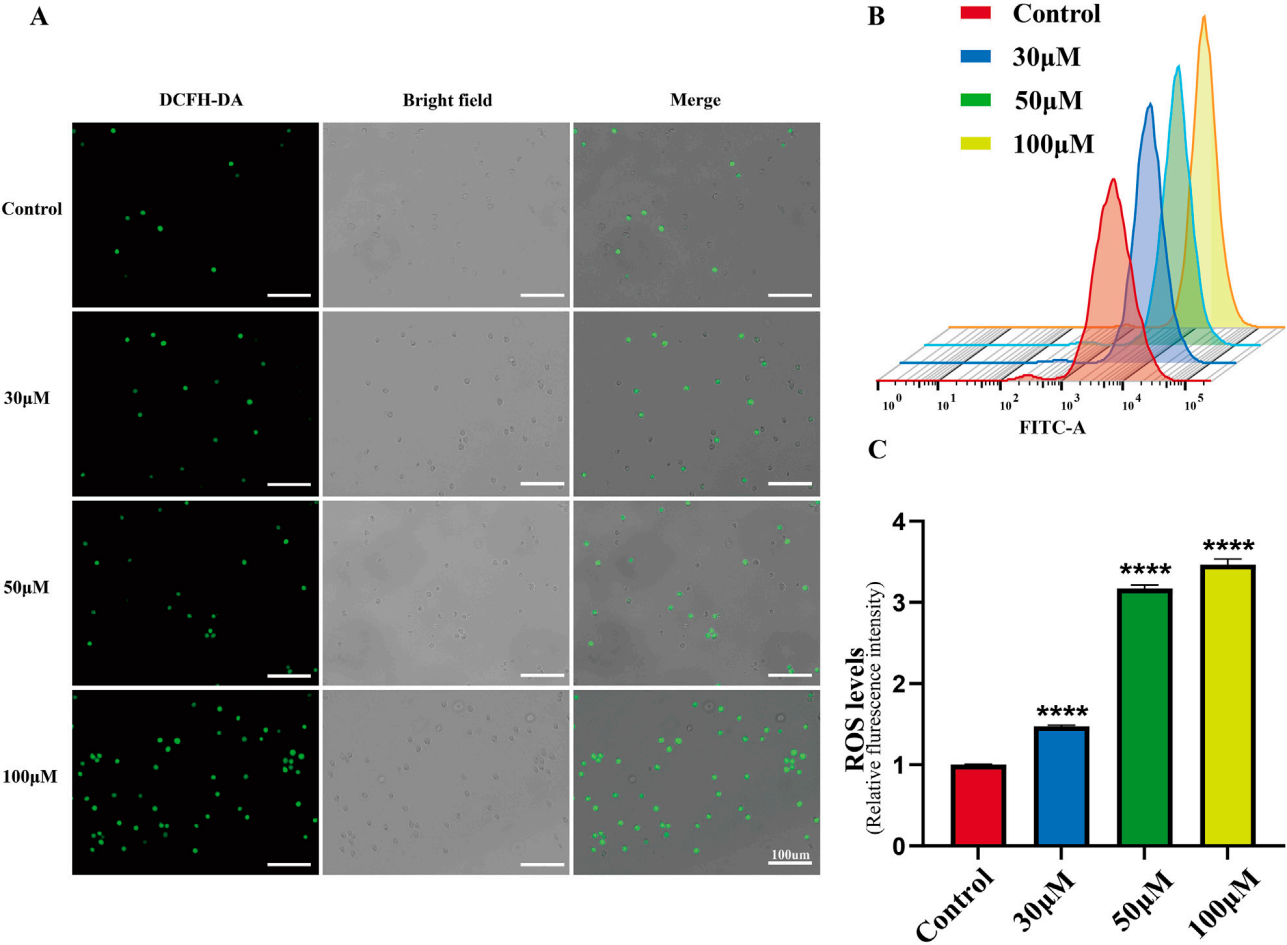
ROS accumulation in SUP-B15 cells induced by luteolin. **(A)** The fluorescence intensity of SUP-B15 cells were visualized under a fluorescence inverted microscope, scale bar = 100 μM. **(B)** Luteolin-induced ROS levels were detected using flow cytometry in SUP-B15 cells. **(C)** Quantitative analysis of ROS levels. Compared with the control group, *p < 0.05, **p < 0.01, ***p < 0.005, ****p < 0.0001.

Excessive ROS production in cells promotes DNA damage and cell death. As a flavonoid compound, luteolin can regulate the redox state of cells. Therefore, we detected ROS accumulation induced by luteolin in SUP-B15 cells using DCFH-DA staining and flow cytometry. After treatment with 0, 30, 50, and 100 μM luteolin for 24 h, the green fluorescence in the luteolin group was significantly increased compared with the blank control group (Figure 7A). As the concentration of luteolin increased, the peak shifted to the right, and the mean fluorescence intensity (MFI) of SUP-B15 cells increased significantly (P < 0.05) (Figures 7B,C). Therefore, it is speculated that ROS plays an important role in luteolin-induced apoptosis of SUP-B15 cells.

### 3.7 Luteolin treatment reduces ATP production and lowers mitochondrial membrane potential

**FIGURE 8:**
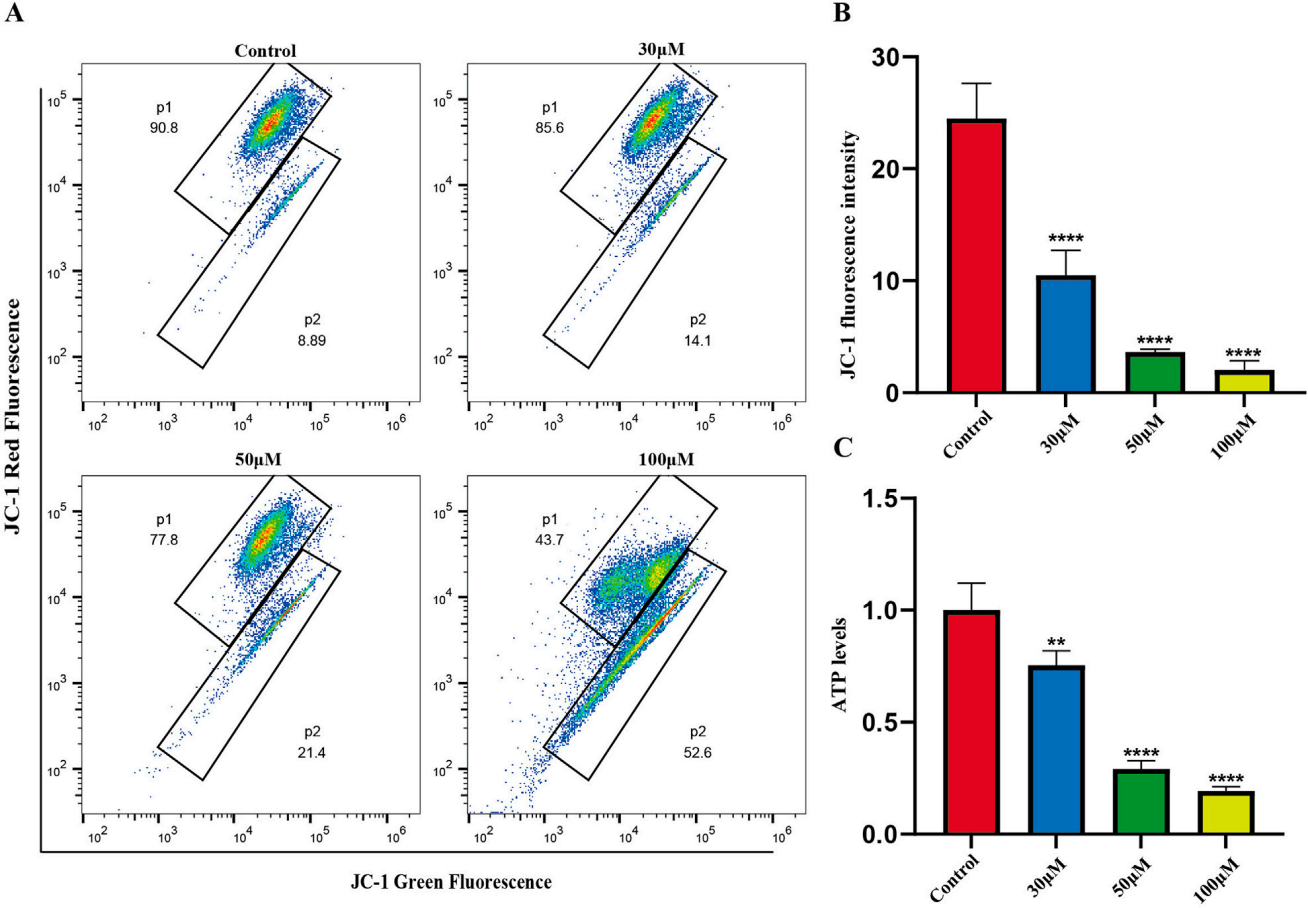
Mitochondrial membrane potential, ATP levels in SUP-B15 cells induced by luteolin. **(A)** The luteolin-treated SUP-B15 cells were stained with JC-1 and analyzed using flow cytometry. **(B)** Quantitative analysis of Mitochondrial membrane potential. Compared with the control group, *p < 0.05, **p < 0.01, ***p < 0.005, ****p < 0.0001. **(C)** The ATP levels were detected based on the microplate system. Compared with the control group, *p < 0.05, **p < 0.01, ***p < 0.005, ****p < 0.0001.

The mitochondrial-dependent apoptosis pathway is triggered by excessive production of reactive oxygen species (ROS). We investigated the effects of luteolin on mitochondrial function in Philadelphia chromosome-positive acute lymphoblastic leukemia cell, including changes in mitochondrial membrane potential and ATP content. Mitochondrial membrane potential was assessed using the JC-1 fluorescent dye. In normal cells, JC-1 accumulates within mitochondria and forms polymers, emitting red fluorescence. In apoptotic or abnormal cells with low membrane potential, JC-1 remains in its monomeric form and emits green fluorescence. This reduction in membrane potential is an early sign of apoptosis. Flow cytometry results showed that treatment of SUP-B15 cells with luteolin at concentrations of 30, 50, and 100 μM significantly increased the red/green fluorescence ratio compared to the blank control group (Figures 8A,B). This indicates that luteolin effectively reduced the mitochondrial membrane potential levels in SUP-B15 cells.

Next, ATP content was measured to assess the effects of luteolin on mitochondrial energy metabolism. The results showed that after treating SUP-B15 cells with luteolin at concentrations of 30, 50, and 100μM, ATP content decreased significantly with increasing drug concentration (Figure 8C), demonstrating that luteolin can significantly reduce ATP levels.

### 3.8 ROS is implicated in the regulation of apoptosis of SUP-B15 cells upon luteolin

**FIGURE 9:**
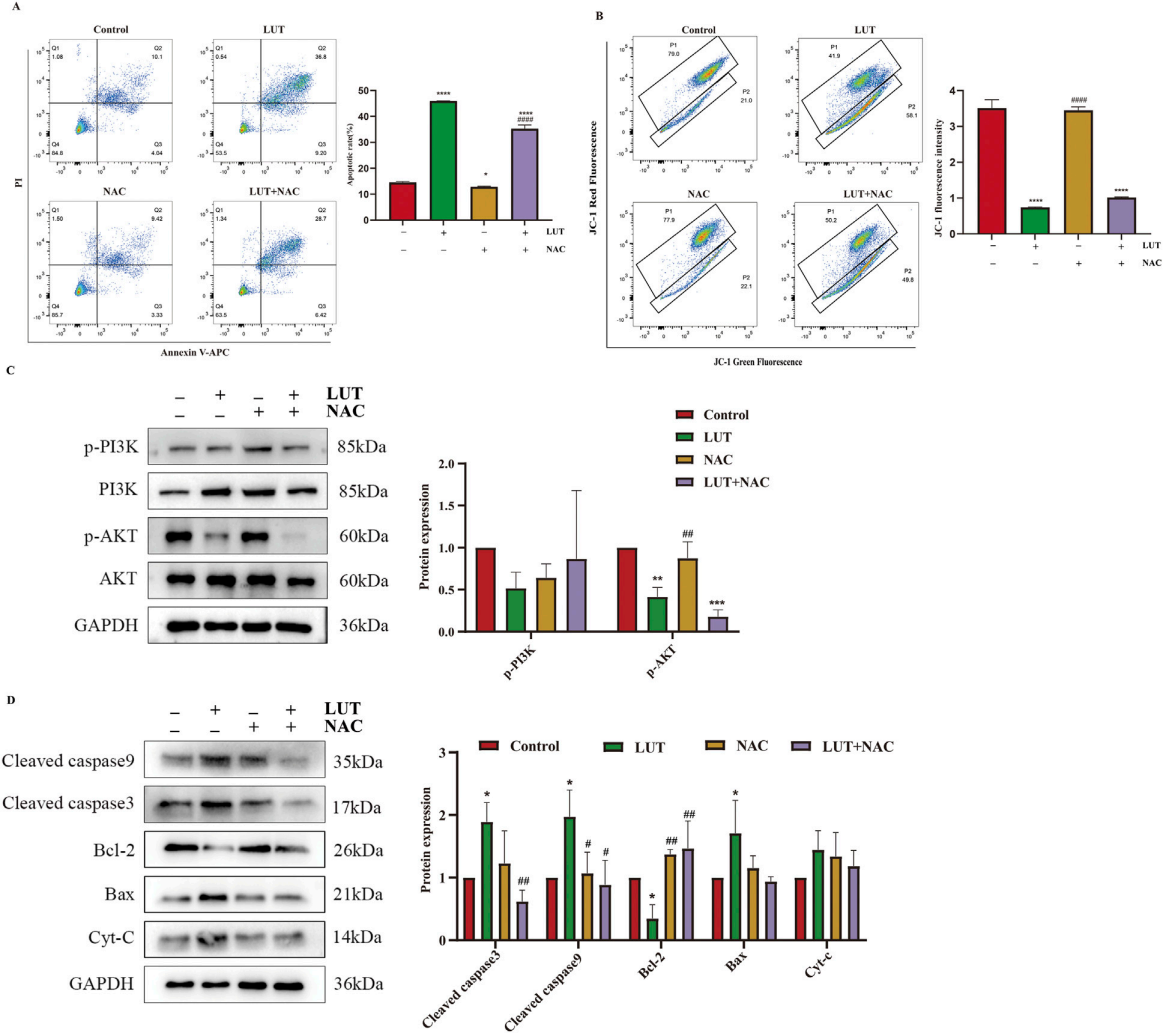
After treatment with luteolin, ROS exerted a pro-apoptotic effect in SUP-B15 cells. The cells were preincubated with 2 mM NAC for 1 h, followed by treatment with 50 μM luteolin for 24 h. **(A)** Flow cytometry analysis of apoptosis. **(B)** Flow cytometry detection of MMP. **(C)** Western blotting was used to detect the expression of proteins related to the PI3K/AKT pathway. **(D)** Western blotting was used to detect the expression of proteins related to the intrinsic apoptosis pathway. Compared with the control group, *p < 0.05,**p < 0.01,***p < 0.005,****p < 0.0001. Compared with the luteolin experimental group, #p < 0.05, ##p < 0.01, ###p < 0.005, ####p < 0.0001.

To determine the role of ROS in the signaling cascade, we pretreated cells with the ROS inhibitor N-acetylcysteine (NAC). We found that NAC partially reversed the apoptosis induction and mitochondrial membrane potential reduction induced by luteolin (Figures 9A,B). Simultaneously, the expression of p-PI3K, p-AKT, BCL-2, BAX, cleaved caspase-3, and cleaved caspase-9 were also eliminated by NAC (Figures 9C,D). In summary, luteolin exerts its pro-apoptotic effects by inhibiting the PI3K/AKT signaling pathway through ROS accumulation.

### 3.9 Luteolin induces apoptosis of SUP-B15 cells by affecting phosphorylation of core pathways and intrinsic apoptotic pathway

**FIGURE 10:**
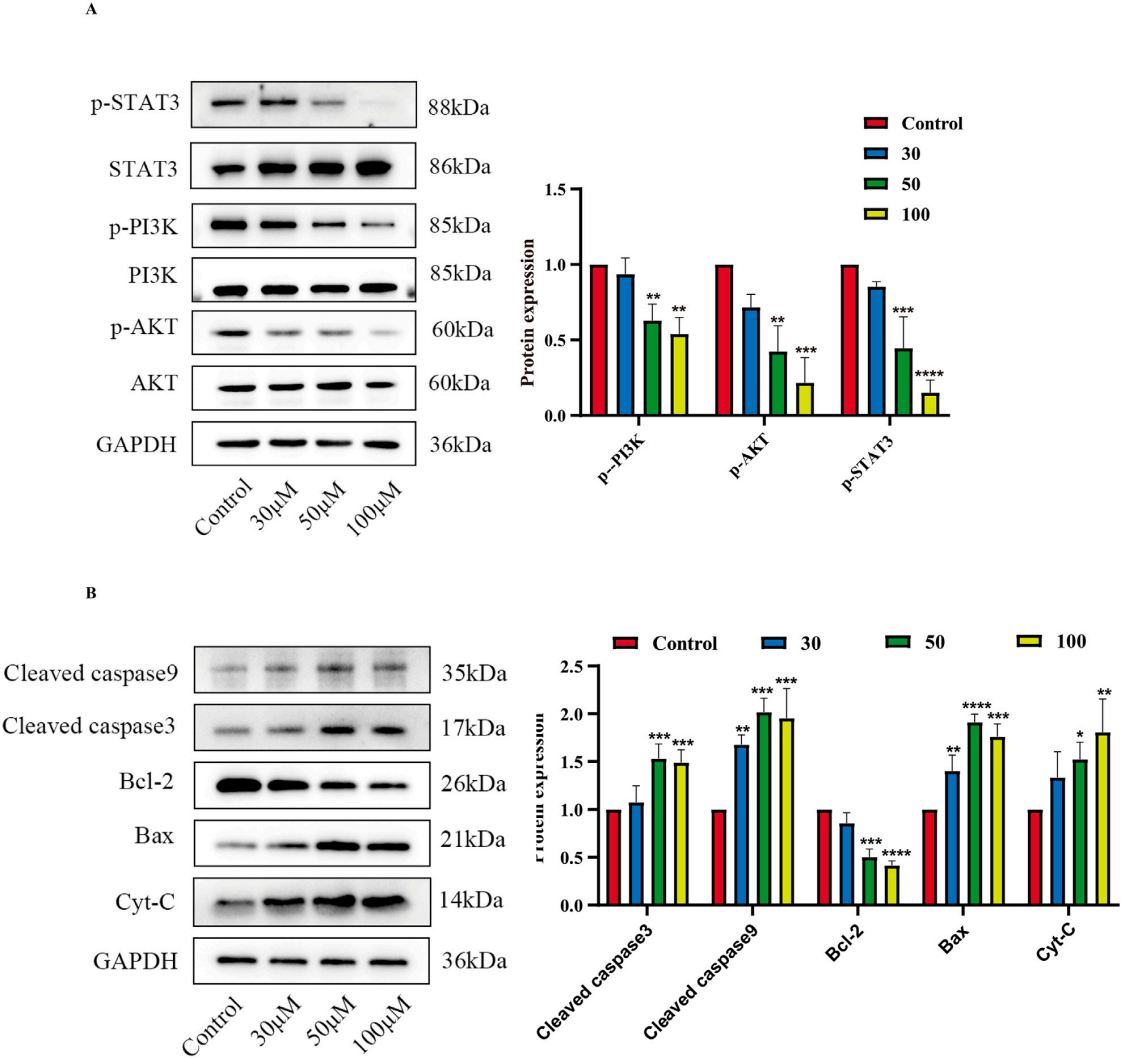
The effects of luteolin on proteins related to the PI3K/AKT pathway, JAK/STAT pathway. **(A)** Treatment with luteolin at concentrations of 0, 30, 50, and 100 μM for 48 h resulted in the expression of proteins related to the PI3K/AKT pathway and STAT3-related proteins. Compared with the control group, *p < 0.05, **p < 0.01, ***p < 0.005, ****p < 0.0001. **(B)** Treatment with luteolin at concentrations of 0, 30, 50, and 100 μM for 48 h resulted in the expression of proteins related to the intrinsic apoptosis pathway. Compared with the control group, *p < 0.05, **p < 0.01, ***p < 0.005, ****p < 0.0001.

Based on the predictions in Section 3.3 above, two core target pathways closely associated with cancer—the PI3K/AKT signaling pathway and the JAK/STAT signaling pathway—were selected to further explore the pro-apoptotic mechanism of luteolin on SUP-B15 cells. RT-qPCR and Western blot were performed to detect PI3K/AKT pathway-related mRNA (Supplementary Figure S1) and protein expression. The results showed that, compared with the control group,the expression levels of p-PI3K, p-AKT, and p-STAT3 were significantly downregulated in the luteolin treatment group. (Figure 10A). Preliminary experiments indicate that luteolin may induce apoptosis in SUP-B15 cells through the mitochondrial-dependent apoptosis pathway. Protein analysis showed that, compared with the control group, the expression levels of pro-apoptotic proteins in the experimental group, including BAX, total cytochrome C, cleaved caspase-9, and cleaved caspase-3, were upregulated, while the expression level of the anti-apoptotic protein BCL-2 was downregulated. (Figure 10B). These results collectively indicate that luteolin may influence the expression of the PI3K/AKT signaling pathway and the JAK/STAT signaling pathway, thereby inducing apoptosis in SUP-B15 cells through the intrinsic apoptotic pathway *in vitro*.

### 3.10 The key role of the PI3K/AKT signaling pathway in luteolin-induced mitochondrial apoptosis

**FIGURE 11:**
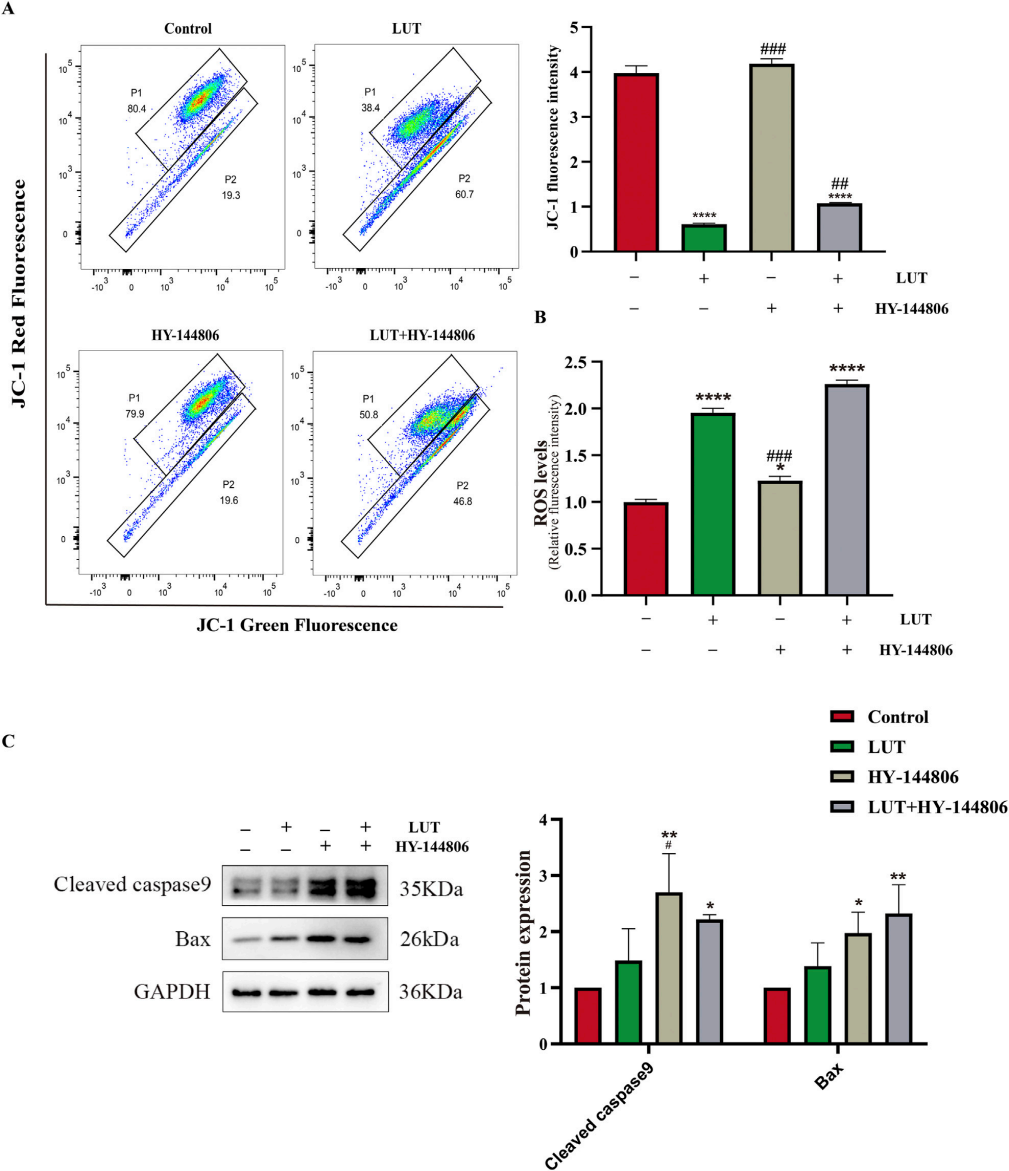
The role of the PI3K/AKT signaling pathway in mitochondrial apoptosis After luteolin treatment. **(A)** MMP detected by flow cytometry. **(B)** ROS detected by a multi-functional microplate reader. **(C)** Western blotting was used to detect the expression of Bax and cleaved caspase-9. Compared with the control group, *p < 0.05, **p < 0.01, ***p < 0.005, ****p < 0.0001. Compared with the luteolin experimental group, #p < 0.05, ##p < 0.01, ###p < 0.005, ####p < 0.0001.

**FIGURE 12:**
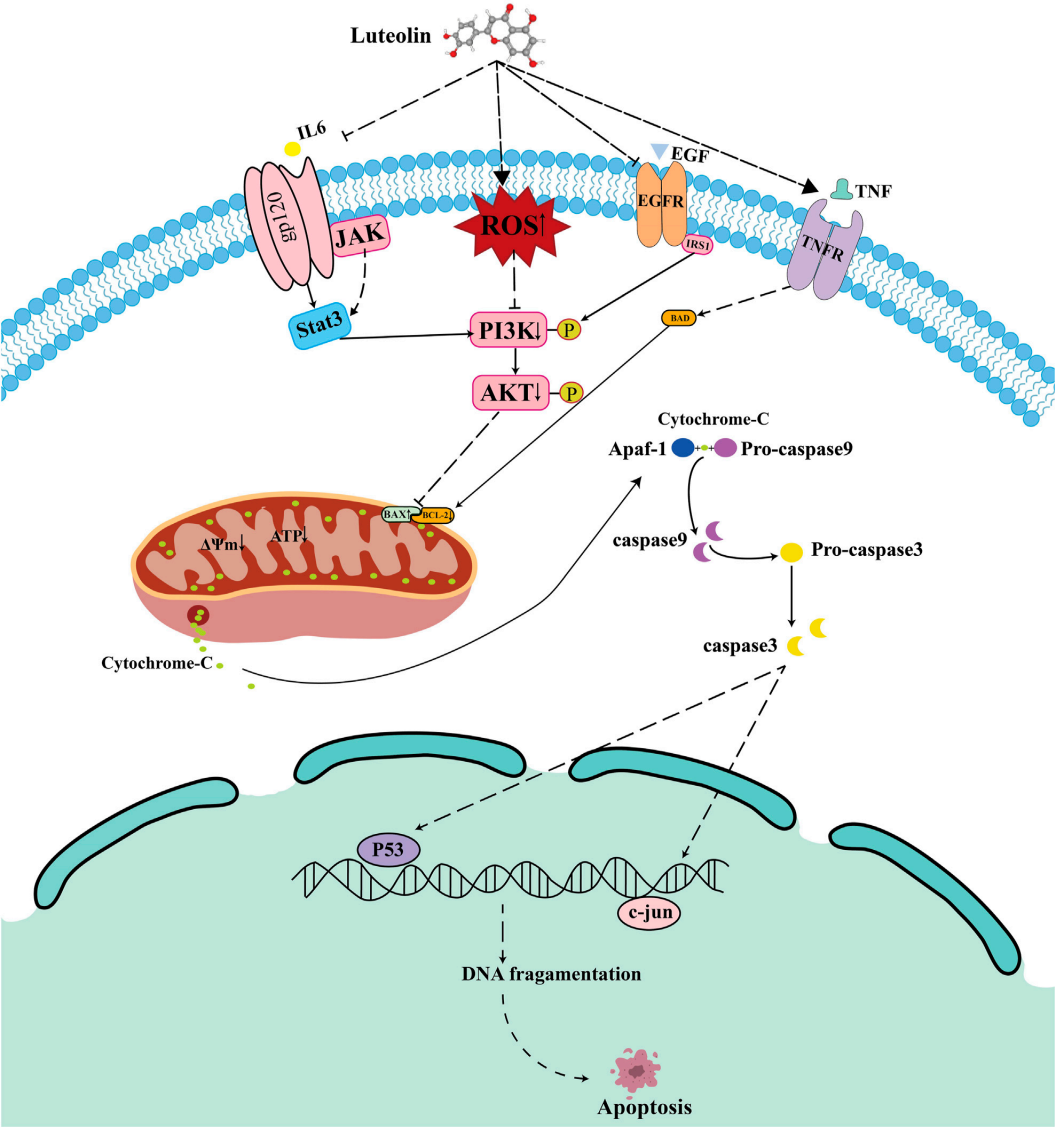
Proposed mechanism of Luteolin-induced apoptosis in Ph + ALL cells.

To elucidate the relationship between PI3K/AKT and mitochondrial apoptosis, we pretreated cells with the PI3K/AKT inhibitor HY-144806. The results revealed that PI3K inhibitors partially recapitulated the effects of luteolin, such as increased ROS levels and upregulation of BAX and cleaved caspase-9 (Figures 11B,C), but the inhibitors did not reduce MMP (Figure 11A). In summary, luteolin may induce mitochondrial apoptosis by inhibiting the PI3K/AKT pathway, but the mitochondrial membrane potential may not be caused by the suppression of the PI3K/AKT signaling pathway. Given the findings in section 3.8 above, the decrease in mitochondrial membrane potential may be caused by the accumulation of ROS. Taken together, these results suggest that luteolin can induce apoptosis in Philadelphia chromosome-positive acute lymphoblastic leukemia cell by inhibiting phosphorylation in the PI3K/AKT signaling pathway (Figure 12).

## 4 Discussion

ALL is characterized by aberrations in the proliferation and differentiation of lymphoblasts, leading to failure of normal immune response and decreased production of normal hematopoiesis (Balsat et al., 2020). Ph + ALL is the most common subtype of B-ALL in adults, accounting for 20%–25% of all genetic subgroups (Chiaretti et al., 2013). Before the introduction of targeted BCR-ABL specific tyrosine kinase inhibitors (TKIs), Adult patients with Philadelphia chromosome-positive acute lymphoblastic leukemia (Ph + ALL) were insufficiently sensitive to standard chemotherapy regimens, resulting in no improvement in overall survival (Faderl et al., 2009). Attempts to improve prognosis through allogeneic stem cell transplantation (SCT) have been only partially successful. Due to the scarcity of sibling donors and the toxic reactions and high mortality associated with SCT, this procedure has failed to produce significant therapeutic effects, making Ph + ALL the most feared subtype of ALL (Maino et al., 2014). The first-generation TKI imatinib marked the beginning of a new era in Ph + ALL treatment, significantly improving the prognosis of Ph + ALL. However, relapse remains a clinical challenge, as it is also associated with drug-resistant substitutions in the ABL kinase domain (Kharas and Fruman, 2005; Thomas and Heiblig, 2016; Kato et al., 2024). Central nervous system (CNS) relapse is also a major challenge in the treatment of Ph + ALL. Poor penetration of imatinib through the blood-brain barrier with inadequate concentrations for kinase inhibition may be associated with an elevated risk of CNS relapse if no additional prophylaxis is given (Takayama et al., 2002; Pfeifer et al., 2003; Gong et al., 2021). Therefore, identifying novel anti-Ph + ALL drugs is of great significance for clinical treatment. It has been discovered that traditional Chinese medicine has proven highly effective in cancer treatment. As extracts from natural products, traditional Chinese medicines offer significant research value in cancer treatment due to their low toxicity and side effects, while also exhibiting inhibitory effects on tumor cells.

Luteolin is a flavonoid compound primarily found in the form of glycosides in plants, the outer shells of legume peanut fruits, and traditional Chinese herbs such as honeysuckle (Singh Tuli et al., 2022; Zhu et al., 2024). Luteolin exhibits pro-apoptotic effects on various cancer cells, with its mechanism of action involving antioxidant activity and excessive ROS production (Kang et al., 2017; Yang et al., 2020; Ma et al., 2023). The objective of this study was to investigate the effects of luteolin on Ph + ALL. Using network pharmacology methods, we identified potential targets for luteolin treatment of Ph + ALL and validated them experimentally. Results indicate that luteolin promotes apoptosis in SUP-B15 cells. This discovery highlights luteolin as a promising therapeutic agent for Ph + ALL, expanding the mechanistic understanding of its anti-leukemic efficacy.

The network pharmacology approach is a new model for the research of traditional Chinese medicine, which aims to predict drug targets and mechanisms of action by constructing a “Drug-disease-target” network. This study employs network pharmacology methodologies to integrate multiple disease databases in order to identify genes associated with Ph + ALL. Ultimately, we identified 154 potential biological targets for luteolin’s action on Ph + ALL. PPI network construction, ten highly central nodes including TP53, AKT1, ALB, TNF, JUN, IL6, EGFR, STAT3, CASP3 and BCL2 were screened from a total of 154 targets of luteolin against the Ph + ALL. TP53 encodes the transcription factor p53, which responds to DNA damage, cellular stress, or oncogenic hyperproliferation (Chen et al., 2022). TP53-mutated ALL is a high-risk disease (Harris et al., 2025). ALB is a parameter for assessing nutritional status and hepatic synthetic capacity. Substantial evidence demonstrates that hypoalbuminemia predicts a poorer prognosis in cancer patients (Zhang et al., 2022). ALB and TNF may serve as potential biomarkers for the diagnosis and treatment of acute lymphoblastic leukemia in children with MLL gene rearrangements (Zhang et al., 2019). The multifunctional cytokine TNF-α, a member of the tumor necrosis factor family, plays a central role in regulating inflammation, immunity, apoptosis, and hematopoiesis. TNF-α correlates with a high-risk disease status in adult B-ALL (Abdalhabib et al., 2022). TNF-α is involved in the progression and recurrence of acute leukemia. Monitoring TNF-α levels may be beneficial for patients with acute leukemia (Verma et al., 2022). JUN is a crucial component of Activator Protein 1 (AP-1) and serves as a key transcription factor regulating physiological and pathological processes such as cell survival, proliferation, and differentiation. (Liu et al., 2020). validated c-JUN as a downstream oncogene of PI3K/AKT, which regulates tumor migration, invasion, and metastasis by binding to genes upstream of PI3K/AKT signaling. IL-6 mediates key interactions in the tumor microenvironment that drive the progression of multiple cancers. The BCR/ABL fusion protein induces IL-6 expression in leukemia stem cells, and targeting IL-6R induces apoptosis in Philadelphia chromosome-positive acute lymphoblastic leukemia cells (Jiang et al., 2018). EGFR is a transmembrane receptor tyrosine kinase that regulates fundamental cellular functions, including proliferation and migration. EGFR activation stimulates the PI3K/Akt pathway, thereby contributing to the molecular pathogenesis of diverse cancers. As these pathways are frequently mutated in T-ALL cells, they represent critical therapeutic targets for this leukemia (Banerjee et al., 2016). Luteolin may influence the progression of Ph + ALL by targeting the aforementioned molecular targets. KEGG pathway analysis indicates that PI3K/AKT signaling pathway and janus kinase/signal transducer and activator of transcription (JAK/STAT) signaling pathway may be essential mechanisms for combating Ph + ALL. The PI3K/AKT signaling pathway plays a central role in the entirety of leukemia pathogenesis, including cell proliferation, transformation, and extramedullary infiltration, making it a crucial therapeutic target (Lim et al., 2022; Cardoso et al., 2023). In drug-resistant esophageal cancer cells, luteolin functions to suppress the FAK/PI3K/AKT pathway, thereby sensitizing them to chemotherapeutic agents (Yang et al., 2024). The PI3K/AKT pathway, located downstream of BCR-ABL1, plays a vital role in BCR-ABL1-mediated leukemia development (Cimino et al., 2006). Constitutive activation of this pathway has been demonstrated to be associated with the development of TKI resistance in cells expressing BCR-ABL1 (Xing et al., 2012). STAT3, a member of the JAK/STAT family of proteins, is in-volved in regulating growth factors and a variety of cytokines. STAT3 plays a crucial role in the survival of ALL cells (Adamaki et al., 2015; Agashe et al., 2022; Wang et al., 2024). Previous research has also confirmed that STAT3 is a potential diagnostic biomarker and therapeutic target for ALL (Zhao et al., 2021). Targeted inhibition of the JAK/STAT3 pathway has emerged as a promising therapeutic strategy for ALL (Jasek-Gajda et al., 2020; Bhansali et al., 2021). This suggests that luteolin may inhibit the growth of ALL cells by regulating the JAK/STAT3 signaling pathway (Singh Tuli et al., 2022; Zong et al., 2024). Consistent with previous reports, our findings indicate that luteolin produces synergistic pro-apoptotic signals by downregulating the expression of STAT3 and PI3K/AKT pathway proteins. Notably, our experimental efforts focused on the most prominent PI3K/AKT predicted pathway, and we recognize that the biological functions of other high-value targets are subjects for subsequent studies.

Mitochondria are central to apoptosis. When cells receive stimuli, mitochondrial membrane permeability increases, releasing apoptotic effectors, thereby activating the intrinsic apoptotic pathway within mitochondria (Poltorak, 2022) B-cell lymphoma 2 (Bcl-2) family proteins act as downstream effector molecules of the PI3K/AKT signaling pathway and are also core regulatory factors of the mitochondrial apoptosis pathway (Singh et al., 2019; Glover et al., 2024; Li et al., 2024; Moyer et al., 2025). Based on structural and functional differences, they can be classified into pro-apoptotic proteins (such as Bak and Bax) and anti-apoptotic proteins (such as Bcl-2 and Bcl-xL) (Czabotar et al., 2013; Kaloni et al., 2023). Upon receiving apoptotic signals, the pro-apoptotic factor BAX is activated, leading to increased mitochondrial membrane permeability. The released cytochrome C further activates the Caspase family. Studies have shown that when cells are exposed to external stimuli, luteolin can induce upregulation of Bax expression while downregulating Bcl-2 expression, increasing mitochondrial membrane permeability, thereby activating the caspase family and triggering apoptosis (Chen et al., 2018; Dong et al., 2018; Wang et al., 2019; Ma et al., 2023). Consistent with the above research results, luteolin downregulates Bcl-2 expression in SUP-B15 cells while upregulating Bax and total cytochrome C expression. In addition, luteolin also upregulates the expression levels of cleaved caspase-9 and cleaved caspase-3 proteins. To clarify the relationship between the PI3K/AKT pathway and mitochondrial apoptosis, we employed the PI3K/AKT inhibitor HY-144806. This inhibitor recapitulates the effects of luteolin, leading to increased reactive oxygen species (ROS), reduced mitochondrial membrane potential, and activation of BAX/cleaved caspase-9. Based on the above results, we speculate that luteolin may further activate the mitochondrial intrinsic apoptosis pathway by inhibiting the PI3K/AKT pathway.

ROS is an important molecule that directly participates in mitochondrial function. The accumulation of ROS leads to changes in mitochondrial membrane permeability, thereby reducing the mitochondrial membrane potential (MMP) (Yang et al., 2016; Li et al., 2021). Research reports indicate that reactive oxygen species produced by leukemia cells induce extracellular trap formation and promote the progression of leukemia (Nikitovic-Tzanakaki et al., 2016). In addition, Wang et al. demonstrated that a new compound synthesized by combining luteolin with zinc oxide nanoparticles (ZnO NPs), zinc oxide nanoparticles-luteolin (ZnONPs-Lut), can induce ROS production, thereby inhibiting cell proliferation (Wang et al., 2025). Consistent with the above results, our study found that luteolin increased ROS accumulation in the mitochondria of SUP-B15 cells, reduced the level of MMP, and decreased ATP synthesis, thereby initiating mitochondria-mediated intrinsic apoptosisThe application of ROS scavengers rescued the luteolin-induced decrease in mitochondrial membrane potential, inhibition of the PI3K/AKT pathway, and subsequent apoptosis, thereby establishing ROS generation as a pivotal initiating event in this signaling cascade. However, the sources of ROS increase remain unclear. Future research should explore these sources to add significant value to the interpretation of the underlying mechanisms. Based on the above analysis, we propose a coherent model: luteolin induces an increase in ROS, which in turn inhibits the PI3K/AKT signaling pathway. This inhibition disrupts the BAX, BCL-2 balance, triggers the loss of mitochondrial membrane potential, and activates Caspase-3,9, ultimately leading to cell apoptosis. This process may also affect other core predicted targets, including STAT3, which requires further investigation in future studies.

It is important to consider that the SUP-B15 cell line expresses the BCR-ABL p190 subtype, and its specific genomic background (involving chromosomes 9, 22 and 4) may influence sensitivity to luteolin. Therefore, the findings of this study provide preliminary mechanistic insights specific to the SUP-B15 model and should not be directly extrapolated to all Ph + ALL contexts. Although BCR-ABL p190 and p210 differ in kinase activity and downstream signaling preferences, the core downstream signaling pathways they activate—such as PI3K/AKT and JAK/STAT—overlap significantly (Jiang et al., 2018; Danışman Kalındemirtaş et al., 2019; Shi et al., 2021). To ensure greater rigor in the research, future work should replicate key experiments across cell lines expressing different isomers, primary patient samples, and *in vivo* models to validate the broad applicability of the findings.

Additionally, the oral bioavailability of luteolin is low and its distribution within tissues is limited. As a microenvironment rich in hematopoietic and immune cells, bone marrow possesses unique physiological barriers—such as the blood-marrow barrier—that restrict the entry of hydrophobic compounds. Therefore, high concentrations of luteolin (100 μM)can only be achieved *in vitro* experiments, which may pose an obstacle to clinical development and application. The development of nanocarrier systems (such as liposomes, nanoparticles, and microemulsions) has significantly enhanced the bioavailability of luteolin (Miyashita et al., 2022; Xu et al., 2023).

In summary, luteolin effectively inhibits the proliferation of SUP-B15 cells, with its efficacy fluctuating over time. This phenomenon may be related to pharmacokinetics and complex multi-target interactions. Furthermore, this study reveals the complexity of the interaction between luteolin and imatinib, which does not necessarily exhibit synergistic effects at all concentrations. It is possible that imatinib triggers compensatory or negative feedback survival signals through other pathways not detected in our study (such as the MAPK or JAK/STAT pathways), thereby partially counteracting the pro-apoptotic signals driven by luteolin. Future studies should employ established models (such as the Chou-Talalay combination index method) for formal synergistic analysis, quantitatively determining the nature of interactions (synergistic, additive or antagonistic) across different concentration ranges, thereby providing direction for subsequent mechanism exploration. The limitations of this study are that the drugs and disease targets are from public database platforms, which may be updated at inconsistent frequencies, and the database data may be incomplete or outdated. Furthermore, the effect and mechanism of luteolin have only been explored in a single cell line. Future studies should incorporate cell lines of different isoforms, primary patient samples, animal experiments and clinical trials to further elucidate the therapeutic effects of luteolin on Ph + ALL. Due to considerations of resources and research focus, this study first conducted experimental validation on the PI3K/AKT pathway—a hub pathway with a higher ranking in the predicted network. However, the other high-priority targets identified in this study require further validation in future research to fully reveal the global landscape of luteolin’s multi-target effects.

## 5 Conclusion

In conclusion, current research indicates that luteolin may induce apoptosis in SUP-B15 cells by inhibiting the PI3K/AKT pathway, potentially accompanied by regulation of other targets such as STAT3. The validation of other predicted core mechanisms and additional high-value targets constitutes an important direction for future investigation. Luteolin demonstrates significant potential as a candidate drug for treating PhALL, offering not only safety and efficacy but also opening new avenues for future drug development and clinical treatment strategies.

## Data Availability

The datasets presented in this study can be found in online repositories. The names of the repository/repositories and accession number(s) can be found below: ren, qin (2025), “Ph+ALL and Luteolin”, Mendeley Data, V1, doi: 10.17632/xr3pd87gpd.1 (https://data.mendeley.com/datasets/xr3pd87gpd/1).

## References

[B1] Abdalhabib E. K. Algarni A. Saboor M. Alanazi F. Ibrahim I. K. Alfeel A. H. (2022). Association of TNF–α rs1800629 with adult acute B-Cell lymphoblastic leukemia. Genes 13 (7), 1237. 10.3390/genes13071237 35886021 PMC9320751

[B2] Adamaki M. Tsotra M. Vlahopoulos S. Zampogiannis A. Papavassiliou A. G. Moschovi M. J. L. R. (2015). STAT transcript levels in childhood acute lymphoblastic leukemia: STAT1 and STAT3 transcript correlations. Leuk. Res. 39 (11), 1285–1291. 10.1016/j.leukres.2015.09.004 26385310

[B3] Agashe R. P. Lippman S. M. Kurzrock R. (2022). JAK: not just another kinase. Mol. Cancer Ther. 21 (12), 1757–1764. 10.1158/1535-7163.Mct-22-0323 36252553 PMC10441554

[B4] Al-Rawashde F. Wan Taib W. R. Ismail I. Johan M. F. Al-Wajeeh A. Al-Jamal H. (2021). Thymoquinone induces downregulation of BCR-ABL/JAK/STAT pathway and apoptosis in K562 leukemia cells. Asian Pac. J. Cancer Prev. 22 (12), 3959–3965. 10.31557/apjcp.2021.22.12.3959 34967577 PMC9080382

[B5] Balsat M. Cacheux V. Carre M. Tavernier-Tardy E. Thomas X. (2020). Treatment and outcome of Philadelphia chromosome-positive acute lymphoblastic leukemia in adults after relapse. Expert Rev. Anticancer Ther. 20 (10), 879–891. 10.1080/14737140.2020.1832890 33016157

[B6] Banerjee K. Das S. Sarkar A. Chatterjee M. Biswas J. Choudhuri S. K. (2016). A copper chelate induces apoptosis and overcomes multidrug resistance in T-cell acute lymphoblastic leukemia through redox imbalance and inhibition of EGFR/PI3K/Akt expression. Biomed. and Pharmacother. 84, 71–92. 10.1016/j.biopha.2016.08.056 27639544

[B7] Bhansali R. S. Rammohan M. Lee P. Laurent A. P. Wen Q. Suraneni P. (2021). DYRK1A regulates B cell acute lymphoblastic leukemia through phosphorylation of FOXO1 and STAT3. J. Clin. Investigation 131 (1), e135937. 10.1172/jci135937 33393494 PMC7773384

[B8] Burmeister T. Schwartz S. Bartram C. R. Gökbuget N. Hoelzer D. Thiel E. (2008). Patients' age and BCR-ABL frequency in adult B-precursor ALL: a retrospective analysis from the GMALL study group. Blood 112 (3), 918–919. 10.1182/blood-2008-04-149286 18650471

[B9] Canichella M. de Fabritiis P. (2025). Tyrosine kinase inhibitor post-allogeneic stem cell transplantation in adult philadelphia-Positive B-Acute lymphoblastic leukemia: state of the art and future directions. Curr. Issues Mol. Biol. 47 (2), 129. 10.3390/cimb47020129 39996850 PMC11854678

[B10] Cardoso B. A. Duque M. Gírio A. Fragoso R. Oliveira M. L. Allen J. R. (2023). CASZ1 upregulates PI3K-AKT-mTOR signaling and promotes T-cell acute lymphoblastic leukemia. Haematologica 109 (6), 1713–1725. 10.3324/haematol.2023.282854 38058200 PMC11141679

[B11] Chai Y. Sun X. Zhou Q. Li H. Xi Y. (2024). Exploration of the mechanism of fraxetin in treating acute myeloid leukemia based on network pharmacology and experimental verification. Heliyon 10 (15), e34717. 10.1016/j.heliyon.2024.e34717 39166080 PMC11334658

[B12] Chen P.-Y. Tien H.-J. Chen S.-F. Horng C.-T. Tang H.-L. Jung H.-L. (2018). Response of Myeloid leukemia cells to Luteolin is modulated by differentially expressed pituitary tumor-transforming gene 1 (PTTG1) oncoprotein. Int. J. Mol. Sci. 19 (4), 1173. 10.3390/ijms19041173 29649138 PMC5979486

[B13] Chen X. Zhang T. Su W. Dou Z. Zhao D. Jin X. (2022). Mutant p53 in cancer: from molecular mechanism to therapeutic modulation. Cell Death and Dis. 13 (11), 974. 10.1038/s41419-022-05408-1 36400749 PMC9674619

[B14] Chiaretti S. Vitale A. Cazzaniga G. Orlando S. M. Silvestri D. Fazi P. (2013). Clinico-biological features of 5202 patients with acute lymphoblastic leukemia enrolled in the Italian AIEOP and GIMEMA protocols and stratified in age cohorts. Haematologica 98 (11), 1702–1710. 10.3324/haematol.2012.080432 23716539 PMC3815170

[B15] Cimino G. Pane F. Elia L. Finolezzi E. Fazi P. Annino L. (2006). The role of BCR/ABL isoforms in the presentation and outcome of patients with Philadelphia-positive acute lymphoblastic leukemia: a seven-year update of the GIMEMA 0496 trial. Haematologica 91 (3), 377–380. Available online at: https://pubmed.ncbi.nlm.nih.gov/16531262/. 16531262

[B16] Czabotar P. E. Lessene G. Strasser A. Adams J. M. (2013). Control of apoptosis by the BCL-2 protein family: implications for physiology and therapy. Nat. Rev. Mol. Cell Biol. 15 (1), 49–63. 10.1038/nrm3722 24355989

[B17] Danışman Kalındemirtaş F. Birman H. Candöken E. Bilgiş Gazioğlu S. Melikoğlu G. Kuruca S. (2019). Cytotoxic effects of some flavonoids and imatinib on the K562 chronic myeloid leukemia cell line: data analysis using the combination index method. Balkan Med. J. 36 (2), 96–105. 10.4274/balkanmedj.galenos.2018.2017.1244 30396879 PMC6409953

[B19] Dong W. Lin Y. Cao Y. Liu Y. Xie X. Gu W. (2018). Luteolin induces myelodysplastic syndrome-derived cell apoptosis *via* the p53-dependent mitochondrial signaling pathway mediated by reactive oxygen species. Int. J. Mol. Med. 42, 1106–1115. 10.3892/ijmm.2018.3696 29786746

[B21] Faderl S. Kantarjian H. M. Thomas D. A. Cortes J. Giles F. Pierce S. (2009). Outcome of Philadelphia chromosome-positive adult acute lymphoblastic leukemia. Leukemia and Lymphoma 36 (3-4), 263–273. 10.3109/10428190009148847 10674898

[B22] Gaballa M. R. Banerjee P. Milton D. R. Jiang X. Ganesh C. Khazal S. (2022). Blinatumomab maintenance after allogeneic hematopoietic cell transplantation for B-lineage acute lymphoblastic leukemia. Blood 139 (12), 1908–1919. 10.1182/blood.2021013290 34914826 PMC8952188

[B23] Glover H. L. Schreiner A. Dewson G. Tait S. W. G. (2024). Mitochondria and cell death. Nat. Cell Biol. 26 (9), 1434–1446. 10.1038/s41556-024-01429-4 38902422

[B24] Gong X. Li L. Wei H. Liu B. Zhou C. Zhang G. (2021). A higher dose of dasatinib May increase the possibility of crossing the blood–brain barrier in the treatment of patients with Philadelphia chromosome–positive acute lymphoblastic leukemia. Clin. Ther. 43 (7), 1265–1271.e1. 10.1016/j.clinthera.2021.05.009 34120773

[B26] Harris E. J. Karaoglu D. A. Sukhanova M. Abaza Y. Karantanos T. Eisfeld A.-K. (2025). Clinical and molecular characterization of TP53-mutant acute lymphoblastic leukemia in adults. Blood Cancer J. 15 (1), 138. 10.1038/s41408-025-01350-5 40813576 PMC12354737

[B27] Imran M. Rauf A. Abu-Izneid T. Nadeem M. Shariati M. A. Khan I. A. (2019). Luteolin, a flavonoid, as an anticancer agent: a review. Biomed. and Pharmacother. 112, 108612. 10.1016/j.biopha.2019.108612 30798142

[B28] Jasek-Gajda E. W. A. Jurkowska H. JasiŃSka M. Litwin J. A. Lis G. J. (2020). Combination of ERK2 and STAT3 inhibitors promotes anticancer effects on acute lymphoblastic leukemia cells. Cancer Genomics - Proteomics 17 (5), 517–527. 10.21873/cgp.20208 32859630 PMC7472455

[B29] Jiang T. Chen J. Huang X. B. Li Y. X. Zhong L. (2018). miR-451a induced apoptosis of Philadelphia chromosome-positive acute lymphoblastic leukemia cells by targeting IL-6R. Neoplasma 65 (06), 907–914. 10.4149/neo_2018_180121N44 30334451

[B30] Kaloni D. Diepstraten S. T. Strasser A. Kelly G. L. (2023). BCL-2 protein family: attractive targets for cancer therapy. Apoptosis 28 (1-2), 20–38. 10.1007/s10495-022-01780-7 36342579 PMC9950219

[B31] Kang Z.-J. Liu Y.-F. Xu L.-Z. Long Z.-J. Huang D. Yang Y. (2016). The Philadelphia chromosome in leukemogenesis. Chin. J. Cancer 35 (1), 48. 10.1186/s40880-016-0108-0 27233483 PMC4896164

[B32] Kang K. A. Piao M. J. Ryu Y. S. Hyun Y. J. Park J. E. Shilnikova K. (2017). Luteolin induces apoptotic cell death *via* antioxidant activity in human colon cancer cells. Int. J. Oncol. 51 (4), 1169–1178. 10.3892/ijo.2017.4091 28791416

[B33] Kato K. Takagi S. Takano H. Tsunoda S. Watanabe O. Yamaguchi K. (2024). A case report of a truncated ABL1 mutation in 2 cases with Philadelphia chromosome-positive B cell precursor acute lymphoblastic leukemia. Int. J. Hematol. 119 (2), 205–209. 10.1007/s12185-023-03691-y 38236369

[B34] Kharas M. G. Fruman D. A. (2005). ABL oncogenes and phosphoinositide 3-Kinase: mechanism of activation and downstream effectors. Cancer Res. 65 (6), 2047–2053. 10.1158/0008-5472.Can-04-3888 15781610

[B35] Li L. Wang J. Feng L. Fan J. Wang J. Tan N. (2021). Rubioncolin C, a natural naphthohydroquinone dimer isolated from Rubia yunnanensis, inhibits the proliferation and metastasis by inducing ROS-mediated apoptotic and autophagic cell death in triple-negative breast cancer cells. J. Ethnopharmacol. 277, 114184. 10.1016/j.jep.2021.114184 33961996

[B36] Li J. Sun M. Tang M. Song X. Zheng K. Meng T. (2024). Mechanism of PI3K/Akt‑mediated mitochondrial pathway in obesity‑induced apoptosis (Review). Biomed. Rep. 22 (3), 40. 10.3892/br.2024.1918 39781039 PMC11707581

[B37] Liang G. Zhao J. Dou Y. Yang Y. Zhao D. Zhou Z. (2022). Mechanism and experimental verification of Luteolin for the treatment of osteoporosis based on network pharmacology. Front. Endocrinol. 13, 866641. 10.3389/fendo.2022.866641 35355555 PMC8959132

[B38] Lim F. Q. Chan A.S.-Y. Yokomori R. Huang X. Z. Theardy M. S. Yeoh A. E. J. (2022). Targeting dual oncogenic machineries driven by TAL1 and PI3K-AKT pathways in T-cell acute lymphoblastic leukemia. Haematologica 108 (2), 367–381. 10.3324/haematol.2022.280761 36073513 PMC9890034

[B39] Liu C. Peng X. Li Y. Liu S. Hou R. Zhang Y. (2020). Positive feedback loop of FAM83A/PI3K/AKT/c-Jun induces migration, invasion and metastasis in hepatocellular carcinoma. Biomed. and Pharmacother. 123, 109780. 10.1016/j.biopha.2019.109780 31901550

[B40] Ma J. Pan Z. Du H. Chen X. Zhu X. Hao W. (2023). Luteolin induces apoptosis by impairing mitochondrial function and targeting the intrinsic apoptosis pathway in gastric cancer cells. Oncol. Lett. 26 (2), 327. 10.3892/ol.2023.13913 37415631 PMC10320424

[B41] Maino E. Sancetta R. Viero P. Imbergamo S. Scattolin A. M. Vespignani M. (2014). Current and future management of Ph/BCR-ABL positive ALL. Expert Rev. Anticancer Ther. 14 (6), 723–740. 10.1586/14737140.2014.895669 24611626

[B42] Miyashita A. Ito J. Parida I. S. Syoji N. Fujii T. Takahashi H. (2022). Improving water dispersibility and bioavailability of luteolin using microemulsion system. Sci. Rep. 12 (1), 11949. 10.1038/s41598-022-16220-4 35831358 PMC9279404

[B43] Moyer A. Tanaka K. Cheng E. H. (2025). Apoptosis in cancer biology and therapy. Annu. Rev. Pathology-Mechanisms Dis. 20, 303–328. 10.1146/annurev-pathmechdis-051222-115023 39854189

[B44] Nikitovic-Tzanakaki D. Yoo H. J. Lee J.-S. Kim J.-E. Gu J. Koh Y. (2016). Extracellular histone released from leukemic cells increases their adhesion to endothelium and protects them from spontaneous and chemotherapy-induced leukemic cell death. Plos One 11 (10), e0163982. 10.1371/journal.pone.0163982 27706246 PMC5051947

[B46] Pfeifer H. Wassmann B. Hofmann W. K. Komor M. Scheuring U. Brück P. (2003). Risk and prognosis of central nervous system leukemia in patients with Philadelphia chromosome-positive acute leukemias treated with imatinib mesylate. Clin. Cancer Res. 9 (13), 4674–4681. Available online at: https://pubmed.ncbi.nlm.nih.gov/14581336/. 14581336

[B47] Poltorak A. (2022). Cell death: all roads lead to mitochondria. Curr. Biol. 32 (16), R891–R894. 10.1016/j.cub.2022.07.025 35998601

[B49] Shi Y.-F. Huang Z.-Y. Huang Y.-S. Dong R.-J. Xing C.-Y. Yu K. (2021). Interaction between CD9 and PI3K-p85 activates the PI3K/AKT signaling pathway in B-lineage acute lymphoblastic leukemia. Oncol. Rep. 46 (1), 140. 10.3892/or.2021.8091 34036396

[B50] Singh R. Letai A. Sarosiek K. (2019). Regulation of apoptosis in health and disease: the balancing act of BCL-2 family proteins. Nat. Rev. Mol. Cell Biol. 20 (3), 175–193. 10.1038/s41580-018-0089-8 30655609 PMC7325303

[B51] Singh Tuli H. Rath P. Chauhan A. Sak K. Aggarwal D. Choudhary R. (2022). Luteolin, a potent anticancer compound: from chemistry to cellular interactions and synergetic perspectives. Cancers 14 (21), 5373. 10.3390/cancers14215373 36358791 PMC9658186

[B52] Takayama N. Sato N. O'Brien S. G. Ikeda Y. Okamoto S. i. (2002). Imatinib mesylate has limited activity against the central nervous system involvement of Philadelphia chromosome‐positive acute lymphoblastic leukaemia due to poor penetration into cerebrospinal fluid. Br. J. Haematol. 119 (1), 106–108. 10.1046/j.1365-2141.2002.03881.x 12358909

[B53] Thomas X. Heiblig M. (2016). The development of agents targeting the BCR-ABL tyrosine kinase as Philadelphia chromosome-positive acute lymphoblastic leukemia treatment. Expert Opin. Drug Discov. 11 (11), 1061–1070. 10.1080/17460441.2016.1227318 27548716

[B54] Verma S. Singh A. Yadav G. Kushwaha R. Ali W. Verma S. P. (2022). Serum Tumor necrosis factor-alpha levels in acute leukemia and its prognostic significance. Cureus 14 (5), e24835. 10.7759/cureus.24835 35547942 PMC9090230

[B56] Wang S. Fu L. Wu Y. Xiao H. Wang J. Sun G. (2019). Influence of luteolin on the apoptosis of esophageal cancer Eca109 cells and its mechanism of action. Food Sci. Hum. Wellness 8 (2), 189–194. 10.1016/j.fshw.2019.03.014

[B58] Wang F. Li Y. Yang Z. Cao W. Liu Y. Zhao L. (2024). Targeting IL-17A enhances imatinib efficacy in Philadelphia chromosome-positive B-cell acute lymphoblastic leukemia. Nat. Commun. 15 (1), 203. 10.1038/s41467-023-44270-3 38172124 PMC10764960

[B59] Wang W. Li Z. Lyu C. Wang T. Han C. Cui S. (2025). Mechanism of a novel complex: Zinc oxide nanoparticles-luteolin to promote ferroptosis in Human Acute Myeloid leukemia cells *in vitro* . Int. J. Nanomedicine 20, 4035–4050. 10.2147/ijn.S509007 40191047 PMC11972579

[B61] Wieduwilt M. J. Yin J. Wetzler M. Uy G. L. Powell B. L. Kolitz J. E. (2021). Dasatinib and dexamethasone followed by hematopoietic cell transplantation for adults with Ph-positive ALL. Blood Adv. 5 (22), 4691–4700. 10.1182/bloodadvances.2021004813 34492682 PMC8759134

[B62] Xing H. Yang X. Liu T. Lin J. Chen X. Gong Y. (2012). The study of resistant mechanisms and reversal in an imatinib resistant Ph+ acute lymphoblastic leukemia cell line. Leukemia Res. 36 (4), 509–513. 10.1016/j.leukres.2011.12.018 22285507

[B63] Xu Q.-T. Zhang W.-X. Xu H.-X. Zhang Q.-F. (2023). Fabrication of Luteolin loaded zein-caseinate nanoparticles and its bioavailability enhancement in rats. J. Pharm. Sci. 112 (12), 3056–3066. 10.1016/j.xphs.2023.06.010 37356712

[B64] Yang Y. Karakhanova S. Hartwig W. D'Haese J. G. Philippov P. P. Werner J. (2016). Mitochondria and mitochondrial ROS in cancer: novel targets for anticancer therapy. J. Cell. Physiology 231 (12), 2570–2581. 10.1002/jcp.25349 26895995

[B65] Yang H. Liu B. F. Xie F. J. Yang W. L. Cao N. (2020). Luteolin induces mitochondrial apoptosis in HT29 cells by inhibiting the Nrf2/ARE signaling pathway. Exp. Ther. Med. 19, 2179–2187. 10.3892/etm.2020.8464 32104282 PMC7027334

[B66] Yang Z. Liu H. Song Y. Gao N. Gao P. Hui Y. (2024). Luteolin enhances drug chemosensitivity by downregulating the FAK/PI3K/AKT pathway in paclitaxel-resistant esophageal squamous cell carcinoma. Int. J. Mol. Med. 54 (3), 77. 10.3892/ijmm.2024.5401 38994756 PMC11265837

[B68] Zhang H. Cheng J. Li Z. Xi Y. (2019). Identification of hub genes and molecular mechanisms in infant acute lymphoblastic leukemia withMLLgene rearrangement. PeerJ 7, e7628. 10.7717/peerj.7628 31523525 PMC6717502

[B69] Zhang Y. Chen Q. Lu C. Yu L. (2022). Prognostic role of controlling nutritional status score in hematological malignancies. Hematology 27 (1), 653–658. 10.1080/16078454.2022.2078040 35622088

[B70] Zhao D. Xing Q. Song H. Zhao Y. Guo G. (2021). LINC00265/miR-4500 axis accelerates acute lymphoblastic leukemia progression by enhancing STAT3 signals. Cancer Manag. Res. 13, 8147–8156. 10.2147/cmar.S274590 34737643 PMC8560060

[B71] Zhu M. Sun Y. Su Y. Guan W. Wang Y. Han J. (2024). Luteolin: a promising multifunctional natural flavonoid for human diseases. Phytotherapy Res. 38 (7), 3417–3443. 10.1002/ptr.8217 38666435

[B72] Zong S. Li X. Zhang G. Hu J. Li H. Guo Z. (2024). Effect of luteolin on glioblastoma's immune microenvironment and tumor growth suppression. Phytomedicine 130, 155611. 10.1016/j.phymed.2024.155611 38776737

