# Supplementary material for: Luteolin induces apoptosis in Philadelphia chromosome-positive acute lymphoblastic leukemia cell by regulating the PI3K/AKT signaling pathway
Source: Front Pharmacol. 2025 Nov 10;16:1676034. doi: 10.3389/fphar.2025.1676034 (PMC12641114; doi:10.3389/fphar.2025.1676034)

**Supplementary Figure S1.** The mRNA levels in SUP-B15 cells after stimulation withluteolin at concentrations of 0, 30, 50, and 100μM for 12 hours. RT-qPCR data are expressed as fold changes relative to the control group, normalized using GAPDH as an internal reference (mean ± standard error, with three replicates per group). *p<0.05, **p<0.01, ***p<0.005, ****p<0.0001 compared with the control group, using one-way analysis of variance.


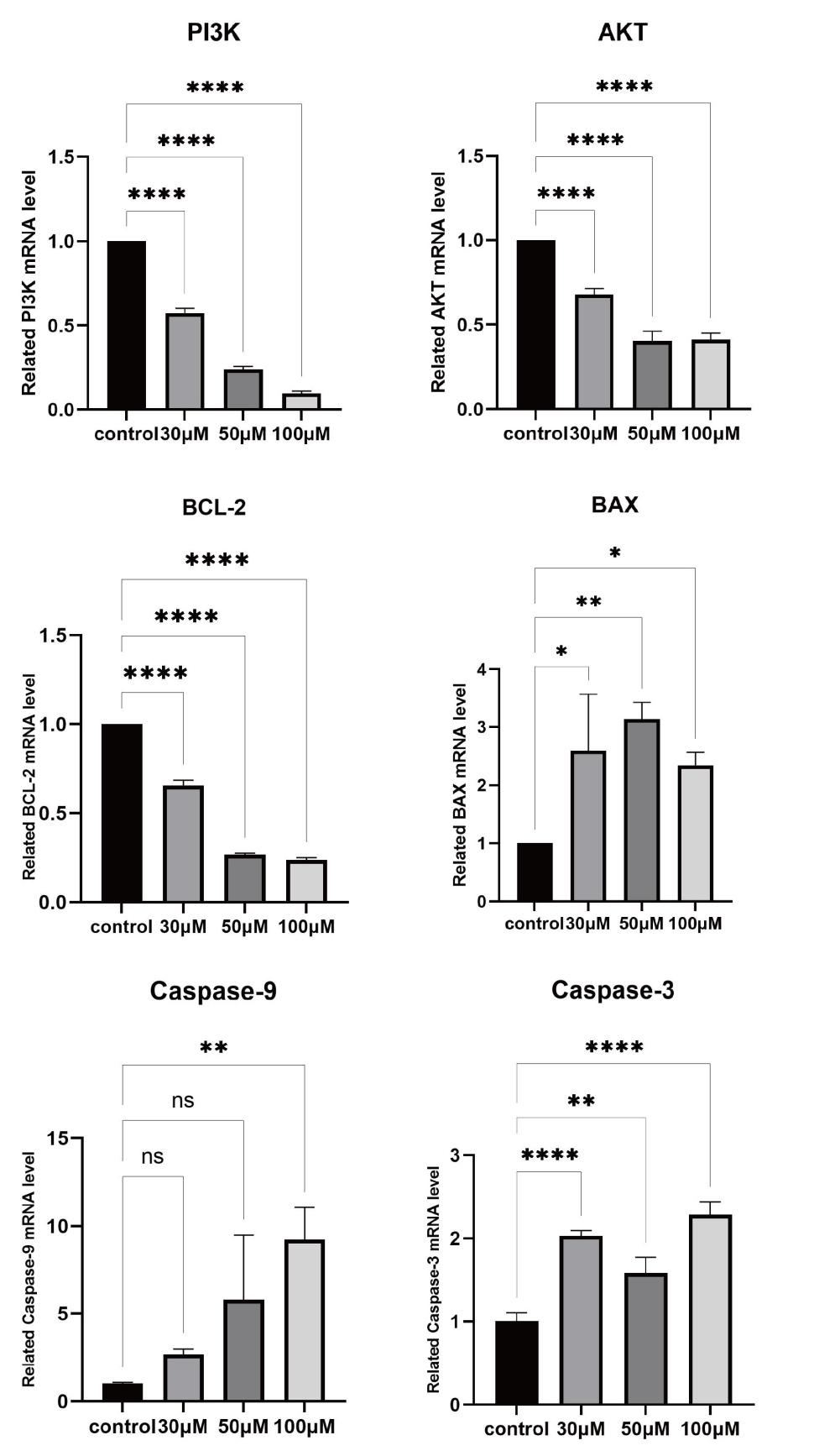

Supplement: Supplementary file 1 [file DataSheet1.zip › Supplementary Material/Supplementary Figure S1.docx]
