# Supplementary material for: Luteolin induces apoptosis in Philadelphia chromosome-positive acute lymphoblastic leukemia cell by regulating the PI3K/AKT signaling pathway
Source: Front Pharmacol. 2025 Nov 10;16:1676034. doi: 10.3389/fphar.2025.1676034 (PMC12641114; doi:10.3389/fphar.2025.1676034)
Supplement: Supplementary file 1 [file DataSheet1.zip › Supplementary Material/Supplementary Table S1.docx]

| Gene | Forward | Reverse |
| --- | --- | --- |
| Bcl-2 | 5'-GGCTGGGATGCCTTTGTG-3' | 5'-CAGCCAGGAGAAATCAAACAGA-3' |
| Bax | 5'-TGCTTCAGGGTTTCATCCAG-3' | 5'-GGCGGCAATCATCCTCTG-3' |
| Caspase-3 | 5'-CAGAACTGGACTGTGGCATTG-3' | 5'-GCTTGTCGGCATACTGTTTCA-3' |
| Caspase-9 | 5'-CCAGAGATTGCGAAACCAGAGG-3' | 5'-GAGCACCGACATCACCAAATTC-3' |
| PI3K | 5'-CGTAGTCGTGATCGGCGATGCA-3' | 5'-CGTAGCTGGATGCTGATGCGAC-3' |
| AKT | 5'-ACCTGATGCTAGTGCCTGATC-3' | 5'-CTAGGGCGTGATGCTGATGCA-3' |
| GAPDH | 5'-ATCATCAGCAATGCCTCC-3' | 5'-CATCACGCCACAGTTTCC-3' |

**Supplementary Table S1**.Primer sequences for quantitative real time-PCR.
